# Synergistic Integration of Artificial Merkel Disc and Meissner Corpuscle via Dermal Papillary Structures for Mechanically Filtered Multimodal Tactile Sensing

**DOI:** 10.1002/advs.76069

**Published:** 2026-06-11

**Authors:** Jaehyeong Kim, Bohee Maeng, Seunghwan Seo, Kyoung‐Yong Chun, Chang‐Soo Han

**Affiliations:** ^1^ School of Mechanical Engineering Korea University Seoul Republic of Korea; ^2^ Center for Somatosensory Molecular‐Level Mimicry Korea University Seoul Republic of Korea

**Keywords:** mechanical filter, mechanoreceptor, multimodal, papillae, surface recognition, tactile sensor

## Abstract

Human skin efficiently perceives tactile stimuli through specialized mechanoreceptors strategically arranged around the papillary structure at the epidermis–dermis junction. Here, we demonstrate a cooperative self‐powered multimodal tactile sensor that mimics both the spatial organization and mechanical functionality of Merkel discs (SA1) and Meissner corpuscles (RA1) within an artificial papillary architecture. The artificial Meissner sensor generates rapid‐adapting responses under slip, while the Merkel sensor produces sustained slow‐adapting outputs under static loading. The modulus contrast between a rigid epidermal layer and a soft dermal layer induces localized stress concentration and, importantly, mechanically filters incoming stimuli by selectively amplifying periodic components. This structural filtering enhances targeted stress delivery and signal amplification compared to sensors without papillary structure, resulting in over 1.5‐fold improvement in pressure sensitivity for Merkel sensor and more than two orders of magnitude enhancement in amplitude with improved frequency‐domain clarity for Meissner sensor. Combined with fingerprint‐inspired microstructures and machine learning, the system achieves 97.5% classification accuracy across 12 fabric–shape combinations and enables tactile regeneration of embossed patterns. This bioinspired platform provides a structural strategy for enhancing multimodal tactile perception in electronic skin and robotics.

## Graphical abstract

A multimodal tactile sensor module that mimics the spatial arrangement and function of Merkel discs and Meissner corpuscles within the human papillary structure operates in a self‐powered manner, responding to both dynamic and static stimuli, achieving tactile perception more similar to human skin.

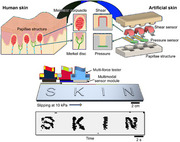

## 1 Introduction

The human tactile system detects a wide variety of mechanical stimuli, including pressure, deformation, shear, and vibration, using specialized mechanoreceptors embedded within the multilayered structure of the skin. Human skin has a layered structure consisting of the epidermis and dermis. Each mechanoreceptor is located regularly at specific locations within the epidermis and dermis, enabling it to detect specific stimuli preferentially. Mechanoreceptors located at the epidermal‐dermal junction—namely Merkel discs (MD, SA1) and Meissner corpuscles (MC, RA1)—exist close to the skin surface. They possess small receptive fields optimized for detecting fine tactile sensations and object features with high resolution [1, 2]. A unique feature of this architecture is the periodic microstructure at the epidermal‐dermal junction, characterized by alternating protrusions and indentations forming a papillary ridge pattern [2]. This papillary (ridge‐and‐valley) microstructure concentrates stress fields at specific locations in response to external mechanical stimuli, enhancing the responses of MD and MC receptors in the vertical and horizontal directions of the skin, respectively. However, while several studies have introduced papillae‐inspired geometries into tactile sensors, the receptor‐specific mechanical role of the papillary ridge–valley structure has not been fully translated into an artificial tactile sensing system.

**FIGURE 1:**
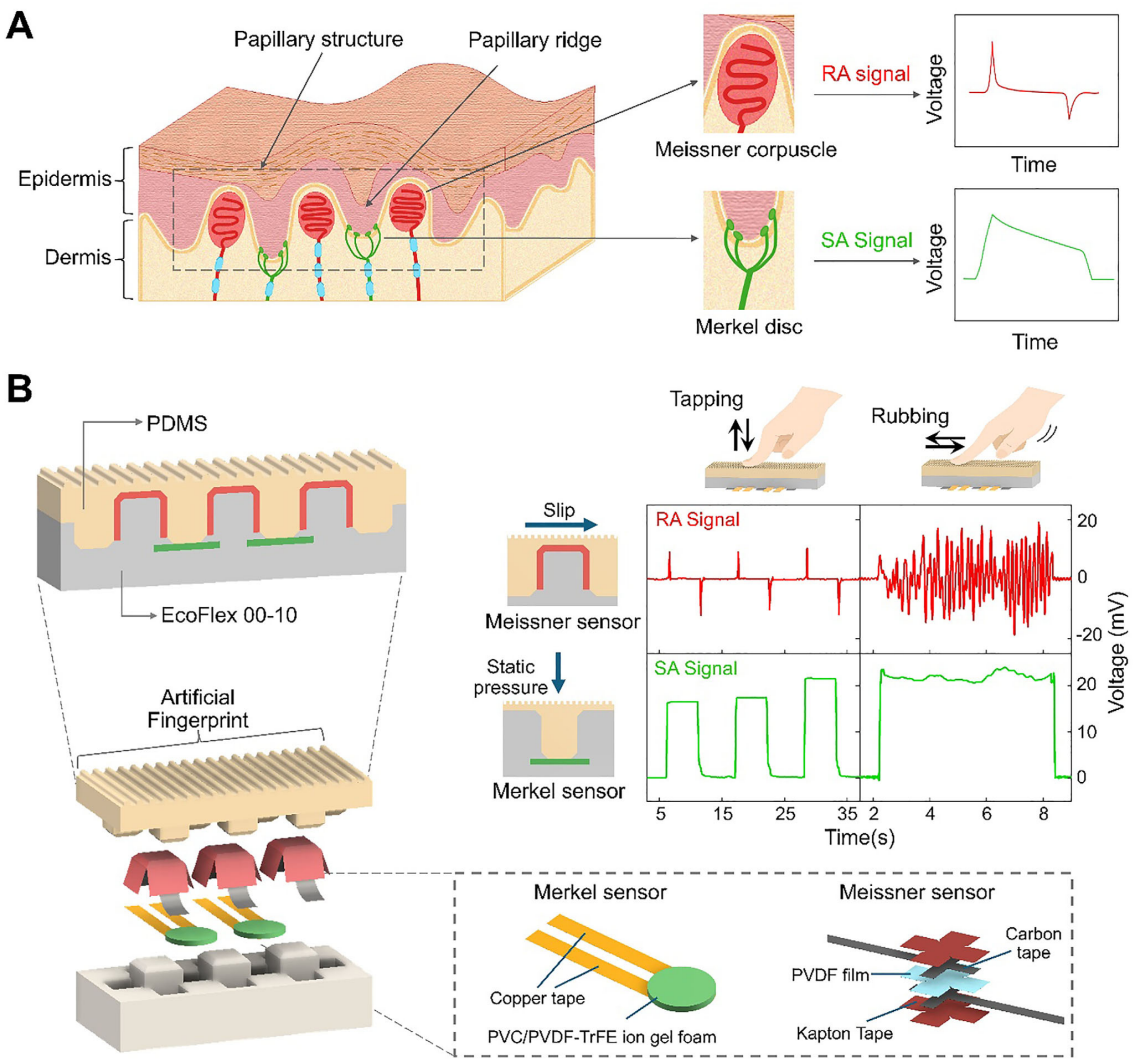
Bioinspired multimodal tactile sensor mimicking papillary structure and RA/SA signal encoding of human skin. (A) Schematic illustration of human skin showing the papillary structure at the dermal–epidermal boundary, with Meissner corpuscles located in the valleys between papillary ridges and Merkel discs positioned directly beneath ridge tips. Representative rapid‐adapting (RA) and slow‐adapting (SA) neural responses from each mechanoreceptor are shown on the right. (B) Our bioinspired tactile sensor mimicking both papillary structure and receptor placement. The epidermis is replicated using PDMS and the dermis using EcoFlex. The Meissner sensor, utilizing cross‐shaped PVDF film, is inserted between papillary ridges to generate RA responses. The Merkel sensor employs a mechanism based on piezoelectric‐induced ion transport to produce SA responses and is positioned directly beneath ridge tips. Representative sensor outputs under tapping and rubbing stimuli are shown, illustrating the functional separation and complementary sensing roles of the two receptors.

As shown in Figure 1A, the MC and MD embedded within this papillary structure perform complementary sensory functions optimized for detecting dynamic (horizontal) and static (vertical) stimuli to the skin, respectively. Located between the papillary ridges near the epidermal interface, the MC acts as a rapid‐adapting (RA) receptor highly sensitive to temporal variations in horizontal skin touch, responding to slippage and transient contact changes. It is particularly responsive to microscopic surface variations, low‐frequency vibrations, and edges [1, 2, 3, 4, 5]. In contrast, MDs are located just beneath the tips of the papillary ridges and function as slow‐adapting (SA) receptors that maintain signal output in response to prolonged mechanical stimuli applied perpendicular to the skin. MDs are specialized in detecting morphological characteristics (contour geometry, curvature profiles, and continuous indentation), enabling shape recognition through continuous pressure [1, 2, 6]. The spatial arrangement of these two receptors enables precise discrimination of both temporal dynamics and spatial information during contact. Furthermore, the fingerprint on the fingertip actively modulates the friction coefficient and strain distribution profile within the contact area, significantly amplifying tactile sensitivity and facilitating the detection of subtle texture variations [2, 7, 8].

Inspired by these biological principles, researchers have pursued the development of flexible artificial sensors that mimic the tactile functions of skin. Various sensing mechanisms, such as resistive, piezoelectric, triboelectric, and capacitive types, have been utilized to detect physical stimuli like normal pressure, tensile strain, tangential shear force, and vibration. However, sensors employing triboelectric or piezoelectric mechanisms are insensitive to static load conditions and respond only to time‐varying inputs [9, 10, 11, 12, 13, 14]. Furthermore, resistive, capacitive [8, 15, 16, 17, 18] and optical [19] sensors require a continuous power supply, making them non‐self‐powered (Table S1). Recent studies have further improved tactile perception through machine learning‐assisted touch modality recognition, dynamic subtle‐stimulus localization, super‐resolution pressure mapping, and inverse design of microstructured tactile sensors [20, 21, 22, 23]. These studies have demonstrated the strong potential of algorithm‐assisted tactile perception and data‐driven sensor optimization. However, they mainly focus on recognition, localization, or performance optimization, rather than hardware‐level decoupling of SA and RA tactile signals. Although simple combinations of sensors can provide stimulus selectivity to some degree, prior studies have not elucidated how each sensor contributes to different components of a complex tactile input. In contrast, our system—informed by the functions of biological SA and RA mechanoreceptors—demonstrates that both sensors can reliably interpret, decompose, and classify multimodal stimuli even when dynamic and static components are simultaneously applied [24, 25, 26, 27, 28].

Several studies have investigated papillae‐inspired geometric features in artificial sensors [29, 30, 31, 32, 33, 34], but most implementations reproduce morphological forms without capturing their functional essence. Papillary patterns have typically been used solely to expand contact surface area, enhancing capacitive sensitivity or detection range, while overlooking the mechanical functionality of papillary structures in natural skin. Consequently, geometric similarity alone is insufficient to reproduce the selective response characteristics exhibited by biological mechanoreceptors. The central innovation of this work is therefore not the simple incorporation of a papillary morphology, but the functional use of the papillary ridge–valley architecture as a receptor‐specific mechanical filtering layer that separates SA and RA tactile pathways before electrical transduction.

This study presents a biomimetic tactile platform that reproduces both the hierarchical structure and functional selectivity inherent in the human skin's papillary structure. Specifically, within the artificial papillary structure, two self‐powered sensors—artificial Meissner and artificial Merkel sensors—are applied at different locations to mimic the structural arrangement and operational characteristics of MC and MD. This artificial papillary structure plays a mechanical filtering role by differentially redistributing stress depending on the type of tactile stimulus, thereby enhancing the performance of the target sensing units. The artificial Meissner sensor positioned between the artificial papillary ridges captures microscopic texture and sliding dynamics, while the artificial Merkel sensor located directly beneath the artificial papillary ridge tips detects sustained pressures, object shape, and curvatures. The artificial Meissner sensor utilizes piezoelectric material, and the artificial Merkel sensor employs the piezoelectric‐iontronic composites [35], achieving complete self‐powered multimodal sensing. To enhance stimulus signal clarity, an artificial fingerprint structure was formed on the artificial epidermis layer. Furthermore, we employed machine learning‐based analysis, including a convolutional neural network (CNN) and t‐distributed stochastic neighbor embedding (t‐SNE), to simultaneously recognize complex tactile inputs composed of both the texture of fabrics and shapes, achieving robust classification across all samples. In addition, we demonstrate that the time domain signals can be converted into spike raster plots inspired by biological neural coding, enabling the system to regenerate embossed characters from scanned tactile inputs into 2D space. The developed sensor not only highlights the importance of papillary structure in tactile perception but will also significantly contribute to future applications of tactile sensors.

## 2 Results and Discussion

### 2.1 Structural Design of Bioinspired Multimodal Sensor

The tactile mechanoreceptors of human skin are allocated in hierarchically organized structures composed of multilayered skin tissue. As shown in Figure 1A, the dermal‐epidermal junction exhibits a papillary structure forming ridges and valleys between the epidermis and dermis. MCs are located in the valleys between papillary ridges, while MDs are positioned just below the tips of the papillary ridges [1, 2, 5]. The cooperative response of these two mechanoreceptors enables sophisticated perception of complex tactile stimuli.

Inspired by this architecture, we designed a multimodal tactile sensor (Figure S1) that mimics both the structural hierarchy and the spatial arrangement of mechanoreceptors within the papillary structure of human skin (Figure 1B). The sensor incorporates an artificial fingerprint structure on the surface, replicated from the geometry of human fingerprints, to enhance tactile sensitivity to surface interactions. The developed sensor consists of a double‐layer structure, with the epidermis made of polydimethylsiloxane (PDMS, base: curing agent ratio 5:1) and the dermis made of EcoFlex 00–10 to have an elastic modulus similar to that of actual human skin [36, 37, 38, 39, 40, 41]. Meissner sensors were fabricated by embedding cross‐shaped polyvinylidene fluoride (PVDF) films coated with carbon tape electrodes and encapsulated by kapton tapes (Figure S2). These films were inserted into the valleys between papillary ridges within the PDMS epidermal layer, mimicking the anatomical placement of MCs between adjacent papillary ridges. In parallel, Merkel sensors were prepared by poly(vinylidene fluoride‐trifluoroethylene) (PVDF‐TrFE)/ polyvinyl chloride (PVC)/dibutyl adipate (DBA)/ 1‐ethyl‐3‐methylimidazolium bis(trifluoromethylsulfonyl)imide (EMIM‐TFSI) composite in tetrahydrofuran (THF) solvent, followed by attachment of copper‐tape electrodes (Figure S3). This Merkel sensor employing piezoelectric‐iontronic effects was positioned directly beneath the papillary ridge tips, analogous to the location of MDs in the dermal region. To verify the contribution of the ionic component to the sustained SA signal, an ionic‐liquid‐free control sensor without EMIM‐TFSI was also tested under the same static loading condition (Figure S4). The control sensor showed only noise‐level output, whereas the EMIM‐TFSI‐containing Merkel sensor produced a sustained SA signal, supporting the role of ion‐mediated polarization in the prolonged static output. This biomimetic assembly enables spatial separation of RA and SA sensing elements, thereby reproducing the dynamic and static response characteristics of human mechanoreceptors.

This spatially separated sensor configuration enables simultaneous yet differentiated responses to distinct tactile stimuli. During static vertical interactions such as tapping, the Merkel sensor generates sustained signals characteristic of slow adapting (SA) mechanoreceptors, whereas during lateral sliding or rubbing motions, the Meissner sensor produces rapid, transient spike‐like responses representative of rapid adapting (RA) mechanoreceptors. When both sensors operate simultaneously, their complementary response patterns allow the system to discriminate and encode static and dynamic tactile information from the same physical interaction. Additional crosstalk experiments further confirmed that these two channels remain functionally separable even under combined normal‐pressure and slip conditions (Figure S5). Specifically, the Merkel channel primarily encoded the sustained normal‐pressure component, whereas the Meissner channel selectively responded to transient pressure changes and slip‐induced periodic vibration. This cooperative yet functionally divided sensing behavior closely replicates the multimodal tactile perception mechanism of human skin.

### 2.2 Mechanical Analysis and Experimental Validation of Papillary Structure

To determine sensor placement and skin materials, finite element analysis (FEA) was performed on a human skin model [3]. This simulation analyzed stress distributions around biologically relevant mechanoreceptors under various loading conditions, considering both the geometry of the papillary structure (Figure S6) and the relative mechanical properties of the epidermis and dermis. To further clarify the functional relevance of this geometry, we also compared the papillary structure with other representative microstructures, including pyramidal and hemispherical structures (Figure S7), to evaluate whether the papillary geometry provides distinct advantages.

**FIGURE 2:**
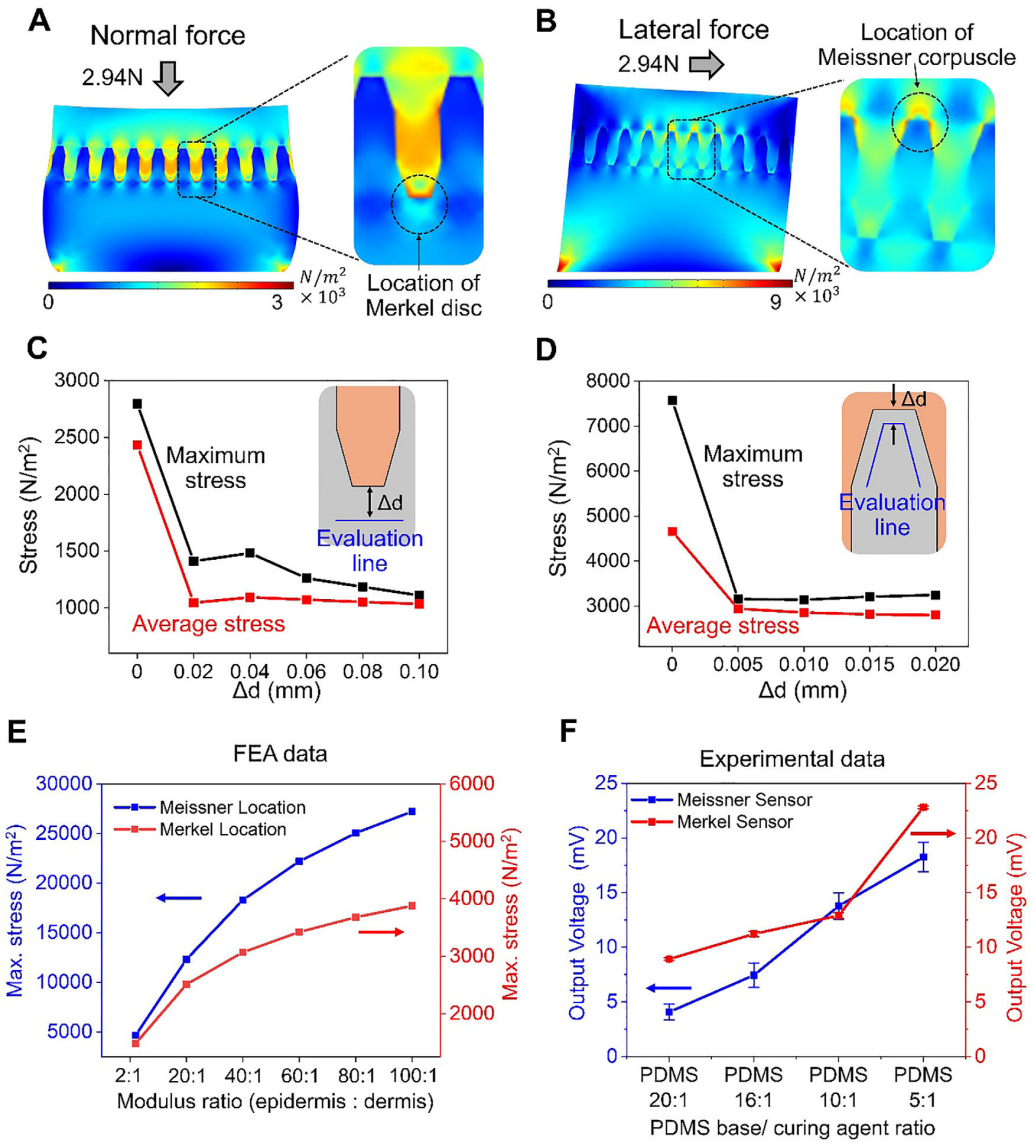
Finite element analysis of geometric and mechanical factors influencing stress concentration within the papillary structure of human skin. (A) FEA results showing stress distribution in a human skin model under 2.94 N normal force, with stress highly concentrated beneath the papillary ridge tip (location of Merkel disc). (B) FEA results under 2.94 N lateral force, showing stress concentration in the valley between papillary ridges (location of Meissner corpuscle). (C) Average and maximum stresses along a virtual evaluation line placed at a vertical distance Δd from the ridge tip. (D) Average and maximum stresses along a virtual evaluation line placed at a horizontal distance Δd from the valley. (E) Maximum stress at the functionally equivalent locations of the Merkel disc (under normal force) and the Meissner corpuscle (under lateral force) as a function of the epidermis‐to‐dermis modulus ratio, obtained from finite element analysis. (F) Experimental data showing the output voltages of the Merkel sensor under normal force test (300 gf) and the Meissner sensor during slip testing (15 mm/s), with a fixed dermis material (Ecoflex 00–10) and varying epidermal PDMS modulus controlled by the base‐to‐curing agent ratio.

Under a normal force of 2.94 N, stress was highly concentrated beneath the papillary ridge tip, matching the anatomical location of MDs (Figure 2A). In contrast, when a lateral force of 2.94 N was applied, stress concentration shifted to the valley region between adjacent papillary ridges along the epidermal–dermal boundary, consistent with the location of MCs (Figure 2B). These results demonstrate that normal and lateral stimuli induce peak stress concentrations at distinct, functionally relevant regions of the papillary structure, corresponding to the anatomical locations of MDs and MCs, respectively. This stimulus‐dependent redistribution of stress indicates that the papillary architecture functions as a passive mechanical filter, selectively routing mechanical energy to specific receptor‐equivalent regions according to the loading condition. Notably, the same stress‐localization tendency was also observed under the low‐load condition of 0.1 N (Figure S8). This result indicates that load scaling changes the absolute stress magnitude but does not alter the spatial stress‐routing pattern of the papillary structure.

To quantitatively analyze stress distributions of these two regions, virtual evaluation lines were placed. For normal loading, the evaluation line was placed vertically at a distance Δd beneath the ridge tip, whereas for lateral loading, the line was drawn horizontally at a distance Δd from the valley region (Figure 2C,D). Along this line, the average and maximum stresses were extracted as functions of Δd. In both cases, the stress values reached their peaks at Δd = 0 mm, indicating that the region immediately beneath the papillary ridge tip under normal loading and the valley region along the epidermal–dermal boundary under lateral force are the mechanically dominant, mechanoreceptor‐equivalent locations. These results explain why the Merkel sensor was positioned directly beneath the artificial papillary ridge tip, while the Meissner sensor was placed along the epidermal–dermal boundary to most effectively capture peak stress concentrations.

Next, the influence of the epidermis‐to‐dermis elastic modulus ratio on stress concentration was examined at both locations. As the modulus ratio increased, the maximum stress at the Merkel disc location under normal force and at the Meissner corpuscle location under lateral force increased monotonically (Figure 2E and Figure S9), indicating that a stiffer epidermis enhances stress transmission to both receptor‐equivalent regions.

To experimentally validate these trends, sensors were fabricated with a fixed dermal layer (EcoFlex 00–10) while varying the epidermal PDMS modulus by adjusting the base‐to‐curing agent ratio, where a lower ratio corresponds to a higher modulus [37, 38]. Under a normal‐force test (300 gf), the output voltage of the Merkel sensor increased as the PDMS ratio decreased. Similarly, during lateral slip tests (15 mm s^−^^1^), the Meissner sensor exhibited higher output voltages as the epidermal modulus increased (Figure 2F). These experimental results closely matched the tendency of FEA results, confirming that modulus difference between epidermis and dermis layers plays a critical role in amplifying stress concentration and sensor sensitivity for both static and dynamic tactile stimuli.

### 2.3 Effect of Artificial Papillary Structure on Mechanically Filtered Tactile Responses

The FEA results show that stress is maximized directly beneath the ridge tips and in the ridge valleys under each stimulus (normal and lateral forces), demonstrating the biomimetic arrangement of Merkel and Meissner sensors. Furthermore, a greater difference in elastic modulus between the epidermis and dermis enhances stress concentration to each mechanoreceptor location, amplifying the output voltage signal. Based on these results, PDMS with a base‐to‐curing agent ratio of 5:1 was selected for the artificial epidermis layer, while EcoFlex 00–10, with a significantly lower elastic modulus, was chosen for the artificial dermis layer. To evaluate how the artificial papillary structure influences tactile signal transmission, the sensing performances of the Merkel and Meissner sensors were compared in configurations with and without papillae. To isolate the structural contribution of the papillary geometry, sensor‐specific comparisons were performed with and without the papillary structure under identical testing conditions (Figure S10).

**FIGURE 3:**
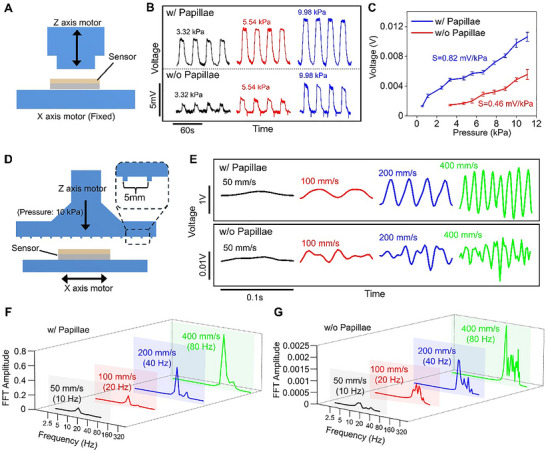
Tactile performance comparison of Merkel and Meissner sensors with and without papillary structures. (A) Schematic of the normal pressure test setup. (B) Representative raw voltage outputs of the Merkel sensor with and without the papillary structure under increasing normal pressures. (C) Comparison of Merkel sensor outputs with (blue) and without (red) papillary structures, showing higher sensitivity (0.82 mV/kPa vs 0.46 mV/kPa) within 0–10 kPa range. Data are presented as mean ± SD from five independently fabricated sensors. (D) Schematic for periodic grating tests (5 mm period) under constant normal pressure (10 kPa) with varying slip speeds. (E) Time‐domain signals during slip over a 5 mm‐pitch grating at different speeds (50–400 mm/s) with and without papillary structure. (F and G) FFT spectra of the corresponding signals with (F) and without (G) papillary structure, displayed on a logarithmic (log_2_) frequency scale. The papillae‐integrated sensor demonstrates more than two orders of magnitude higher amplitude and distinct spectral peaks at the imposed frequencies (10–80 Hz).

To validate the performance of the Merkel sensor and confirm the influence of the artificial papillary structure on performance, vertical pressure tests were conducted (Figure 3A). As shown in Figure 3B, the output voltage increased with increasing applied normal pressure for both configurations, with and without the papillary structure. However, the signal amplitude was consistently larger when the papillary structure was present. As shown in Figure 3C, the sensor with papillary structure exhibited a higher pressure sensitivity (0.82 mV/kPa) compared to the planar configuration without papillae (0.46 mV/kPa). In addition, the operational reliability of the Merkel sensor was examined through long‐term cyclic loading tests and environmental stability tests under different temperature and humidity conditions (Figure S11). The low detection limit was improved in the presence of the papillary structure, indicating enhanced stress concentration beneath the artificial papillary ridge. The measurements were conducted over a pressure range of 0–10 kPa, corresponding to typical contact pressures exerted by a human fingertip during gentle touch [8, 42, 43].

Next, the performance of the Meissner sensor was evaluated. Dynamic slip tests were performed using a periodic grating surface with a 5 mm pitch under a constant normal load of 10 kPa while varying the slip speed from 50 to 400 mm/s (Figure 3D). As shown in Figure 3E, the Meissner sensor integrated within the papillary structure generated stable and well‐defined periodic voltage waveforms whose amplitudes increased with slip speed. Notably, the signal amplitude with papillae was more than two orders of magnitude higher than that of the planar configuration without papillae. In addition to this substantial amplitude enhancement, the papillae‐integrated sensor produced clean and periodic waveforms, whereas the configuration without papillae exhibited irregular, noise‐like signals that partially obscured the imposed periodicity.

To quantitatively analyze frequency responses, FFT analysis was performed (Figure 3F,G). With papillae, sharp spectral peaks precisely matched the imposed frequencies (10–80 Hz), and the signal amplitude increased by more than two orders of magnitude compared to the planar configuration. Without papillae, the spectral peaks were broader and significantly weaker, often obscured by noise. These results demonstrate that the artificial papillary structure functions as a passive mechanical filter, selectively redistributing and amplifying stimulus‐dependent mechanical energy to the Meissner sensor while suppressing non‐periodic disturbances. The inter‐device reproducibility of this dynamic response was further evaluated using five independently fabricated sensors under a representative 10 Hz grating‐slip condition (Figure S12). The papillae‐integrated sensors consistently generated periodic voltage signals and distinct FFT peaks near the imposed frequency, whereas the sensors without papillae showed much weaker and more irregular responses.

Notably, finite element analysis of the actual sensor geometry (Figure S13) confirmed that the papillary structure induces substantially higher and more localized stress at the sensor sites under both normal and lateral loading compared to a planar configuration. This geometry‐driven stress routing provides the mechanical basis for the experimentally observed amplitude enhancement and frequency selectivity. Together, these results establish that the artificial papillary structure not only increases signal amplitude but also mechanically filters tactile stimuli, improving signal clarity and frequency discrimination in multimodal tactile sensing.

### 2.4 Effect of Artificial Fingerprint Structure on Microstructure Discriminability

**FIGURE 4:**
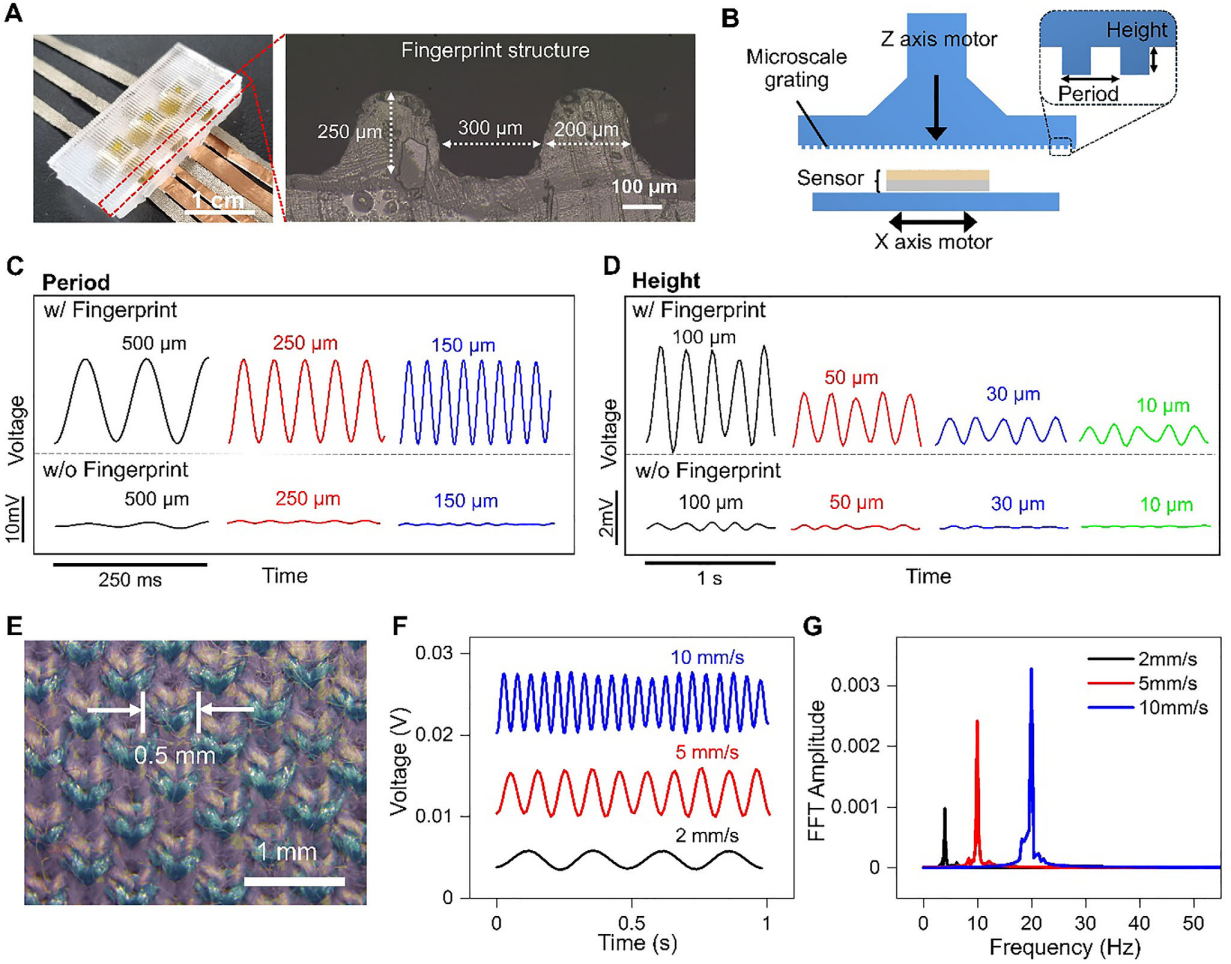
Fingerprint structure enabled enhancement of microstructure discriminability of Meissner sensor. (A) Photograph of the fabricated sensor (left) and cross‐sectional image of the fingerprint structure (right) showing width of 200 µm, distance of 300 µm, and height of 250 µm, mimicking human fingerprint geometry. (B) Schematic of the experimental setup where microscale grating structure was pressed normally onto the sensor with 300 gf and slipped at the speed of 5 mm/s. (C) Output voltage signals obtained from gratings with different periods (500, 250, 150 µm; height fixed at 200 µm). (D) Output voltage signals from gratings with different heights (100, 50, 30, and 10 µm; period fixed at 1 mm, 5 Hz imposed). (E) Image of woven fabric with a characteristic period of 500 µm. (F) Time‐domain signals recorded from the fabric under different sliding speeds (2, 5, and 10 mm/s). (G) FFT spectra corresponding to (F), with distinct peaks at imposed frequencies (4, 10, and 20 Hz).

In biological skin, MCs are located beneath the ridges of fingerprints and align with the periodic geometric structure of the skin surface [1, 2]. This anatomical arrangement suggests that fingerprints play a crucial role in amplifying the dynamic response of MC. Based on this biological evidence, we investigated their role in microstructure and texture recognition by fabricating artificial fingerprint structures on the artificial epidermis surface that mimic the geometric features of human fingerprints [44, 45], thereby significantly enhancing the microstructure discriminability of Meissner sensor. Cross‐sectional images of the fabricated sensor and fingerprint structure are shown in Figure 4A, revealing the continuous ridges and valleys of the fingerprint structure (ridge width: 200 µm, valley spacing: 300 µm, height: 250 µm). The experimental setup is schematically illustrated in Figure 4B, where a microscale grating structure with controlled period and height (Figure S14) was brought into contact with the sensor. The z‐axis motor was fixed at a load of 300 gf, while the x‐axis motor applied lateral sliding at a constant speed of 5 mm/s.

The effect of microstructure discriminability in the horizontal direction was first investigated by fixing the height at 200 µm and varying the grating period from 500 to 150 µm (Figure 4C). The Meissner sensor with the fingerprint structure generated a larger signal amplitude and stably identified the imposed frequency even when the grating period was as small as 150 µm. In contrast, without the fingerprint structure, the signal amplitude decreased sharply, making it impossible to distinguish periodic frequency components, especially for smaller grating periods. FFT analysis confirmed that sharper spectral peaks were observed at the imposed frequency when the fingerprint structure was present (Figure S15).

Next, we evaluated microstructure discriminability in the vertical direction by varying the grid height from 100 to 10 µm – which is similar to human tactile performance [2] – while fixing the period at 1 mm (Figure 4D). A consistent 5 Hz stimulus was applied in all cases. With fingerprint structures present, the sensor generated relatively large signal amplitudes and reliably identified each frequency. However, in the absence of fingerprint structures, the amplitude was significantly lower, and the sensor barely recognized the given frequency at the smallest grid height. This was also confirmed in the FFT spectrum (Figure S16).

Additionally, we examined the surface detection performance by attaching fabric with a characteristic period of approximately 500 µm to the linear tester instead of the grating structure (Figure 4E). When the fabric was brought into contact with the sensor and slid at different speeds of 2, 5, and 10 mm/s, the sensor generated strong periodic responses, with the amplitude increasing as the sliding speed increased (Figure 4F). The corresponding FFT spectrum exhibited sharp peaks at each frequency (4, 10, and 20 Hz) (Figure 4G). These results demonstrate that fingerprint structures play a crucial role in enhancing the microstructure discriminability and frequency selectivity of the Meissner sensor under dynamic sliding conditions.

### 2.5 Cooperative Signal Encoding Through Dual‐Channel Signal Integration

**FIGURE 5:**
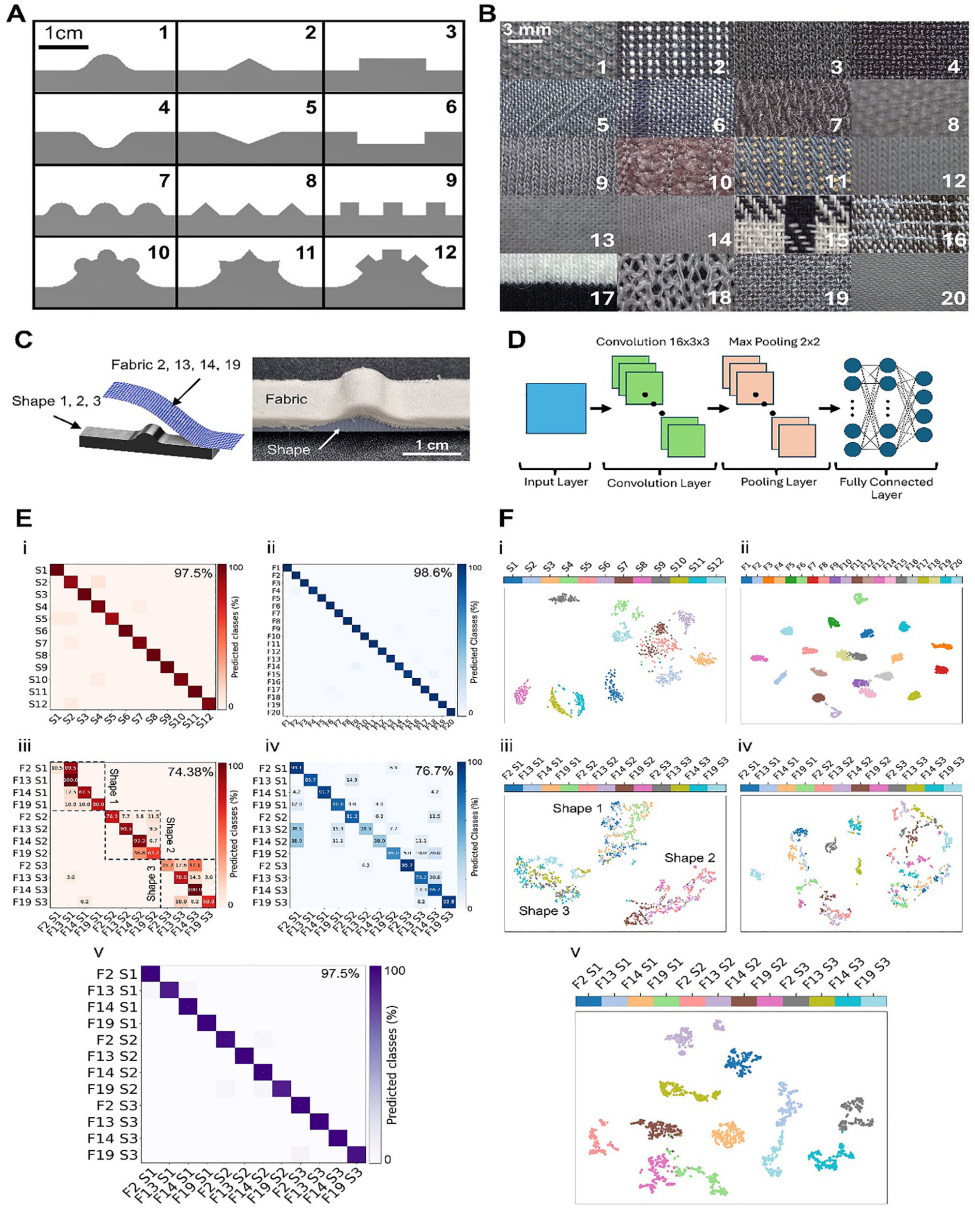
Distinct and complementary recognition of shapes, fabrics, and their combinations by Merkel and Meissner sensors. (A) Twelve centimeter‐scale geometric specimens used for shape recognition experiments. (B) Twenty different fabrics used for fabric recognition experiments. (C) Fabric—shape specimens prepared by laminating four representative fabrics (Nos. 2, 13, 14, 19) onto three shapes (Nos. 1–3), yielding twelve combined stimuli. (D) Schematic of the CNN model used for classification. (E, F) CNN‐based confusion matrix results (E) and t‐SNE feature visualizations (F) for five recognition conditions: (i) shape recognition using Merkel sensor data only, (ii) fabric recognition using Meissner sensor data only, (iii) fabric‐shape recognition using Merkel sensor data only, (iv) fabric‐shape recognition using Meissner sensor data only and (v) fabric‐shape recognition using concatenated Merkel and Meissner sensor data.

Based on scientific evidence that mechanoreceptors within human skin are independently and cooperatively employed to effectively perceive the complicated stimuli from the external environment, we evaluated how artificial Merkel and Meissner sensors differentially and complementarily perceive and classify an object shape and microscopic texture. This ultimately resembles the cooperative encoding strategy of human tactile systems. In this study, we conducted three types of experiments: (i) Shape recognition using 12 geometric objects with distinct shapes (Figure 5A), (ii) Fabric recognition using 20 different fabrics (Figure 5B), and (iii) Fabric‐shape combination stimuli created by overlaying selected fabrics (Fabric 2, 13, 14, 19) onto representative shapes (Shape 1, 2, 3) (Figure 5C). Each sample was slid over the sensor at a constant speed of 5 mm/s. The recorded time domain signals were converted into short‐time Fourier transform (STFT) spectrograms, and sensor performance was evaluated using a simple CNN model and t‐SNE analysis.

The raw time‐domain signals highlighted the modal sensitivity of each sensor. First, the Merkel sensor produced distinct waveform profiles depending on the shape, clearly reflecting differences in geometric shape and curvature (Figure S17). The Meissner sensor exhibited sharp vibration signals when sliding over various fabrics, capturing the periodic microstructure of each fabric sample (Figure S17). These characteristics closely resemble those of biological mechanoreceptors. While MD is sensitive to indentation, shape, and curvature, MC is known to respond to subtle surface features and rapid changes in skin indentation [2].

For quantitative classification, approximately 200 STFT spectrogram samples per class were generated from the time‐domain signals (Figures S18–S21) and fed into a lightweight CNN comprising two convolutional layers and one max‐pooling layer (Figure 5D). This demonstrates that robust classification can be achieved without deep or complex architectures. In this analysis, CNN was used for quantitative classification and accuracy calculation, whereas t‐SNE was used for visualization of feature separability. Accordingly, the CNN‐based confusion matrices are presented in Figure 5E, and the corresponding t‐SNE‐based feature visualizations are presented separately in Figure 5F.

The CNN‐based classification results quantitatively demonstrated the modality‐specific and complementary roles of the Merkel and Meissner sensors. In the shape recognition test, the Merkel sensor achieved a classification accuracy of 97.5% (Figure 5E‐i). In the fabric recognition test using 20 fabrics with fine textures, the Meissner sensor achieved a classification accuracy of 98.6% (Figure 5E‐ii). For the fabric‐shape combination test (Video S1), three data configurations were analyzed: Merkel sensor data only, Meissner sensor data only, and joint Merkel and Meissner sensor data. When only the Merkel sensor data were used, the CNN classification accuracy decreased to 74.38% (Figure 5E‐iii), and the confusion matrix showed that the model often failed to distinguish different fabrics within the same shape category, although it still classified different shapes correctly. This shape‐dominant classification trend was consistently observed across five independently fabricated sensors (Figure S22). When only the Meissner sensor data were used, the CNN classification accuracy was limited to 76.7% (Figure 5E‐iv). Finally, when Merkel and Meissner sensor data were analyzed together, the CNN classification accuracy significantly improved to 97.5% (Figure 5E‐v). To confirm inter‐device reproducibility, the same CNN analysis was repeated using five independently fabricated sensors, and the device‐wise accuracies are summarized as mean ± SD (Figure S22). Across all devices, the combined Merkel and Meissner channels consistently showed higher fabric–shape recognition accuracy than either single channel. These results indicate that the Merkel sensor is specialized for detecting overall shape features, whereas the Meissner sensor contributes to texture‐related dynamic information, and that combining both sensor channels is necessary for robust recognition of combined fabric‐shape stimuli.

The t‐SNE visualization results further supported this interpretation by qualitatively showing feature separability in a 2D space. The Merkel sensor data for shape recognition formed well‐separated groups (Figure 5F‐i), while the Meissner sensor data for fabric recognition also showed clear separation among fabric types (Figure 5F‐ii). In the fabric‐shape combination test, the Merkel‐only features were grouped mainly by shape category rather than by each fabric‐shape pair (Figure 5F‐iii), confirming that the Merkel sensor primarily encodes shape information. The Meissner‐only features showed mixed distributions without complete separation among all fabric‐shape combinations (Figure 5F‐iv), indicating that the Meissner sensor alone is insufficient for classifying combined stimuli. In contrast, when data from both sensors were combined and analyzed, the features formed distinct groups for all texture‐shape combinations (Figure 5F‐v). These t‐SNE results were used only to visualize feature separability and were not used to calculate classification accuracy.

Furthermore, to examine whether this improvement originated from the papillary structure rather than the CNN alone, we performed additional control classification experiments using a sensor without the papillary structure under the same CNN architecture (Figure S23). The control sensor showed lower accuracies, particularly in Meissner‐related recognition tasks, suggesting that the papillary structure improves the quality of dynamic tactile features before CNN classification. Taken together, these results indicate that the Merkel sensor is tuned to shape encoding, the Meissner sensor captures texture microstructure, and the complementary contributions of both modalities are necessary for robust recognition of combined stimuli.

### 2.6 Spatiotemporal Regeneration of Alphabetic Patterns

**FIGURE 6:**
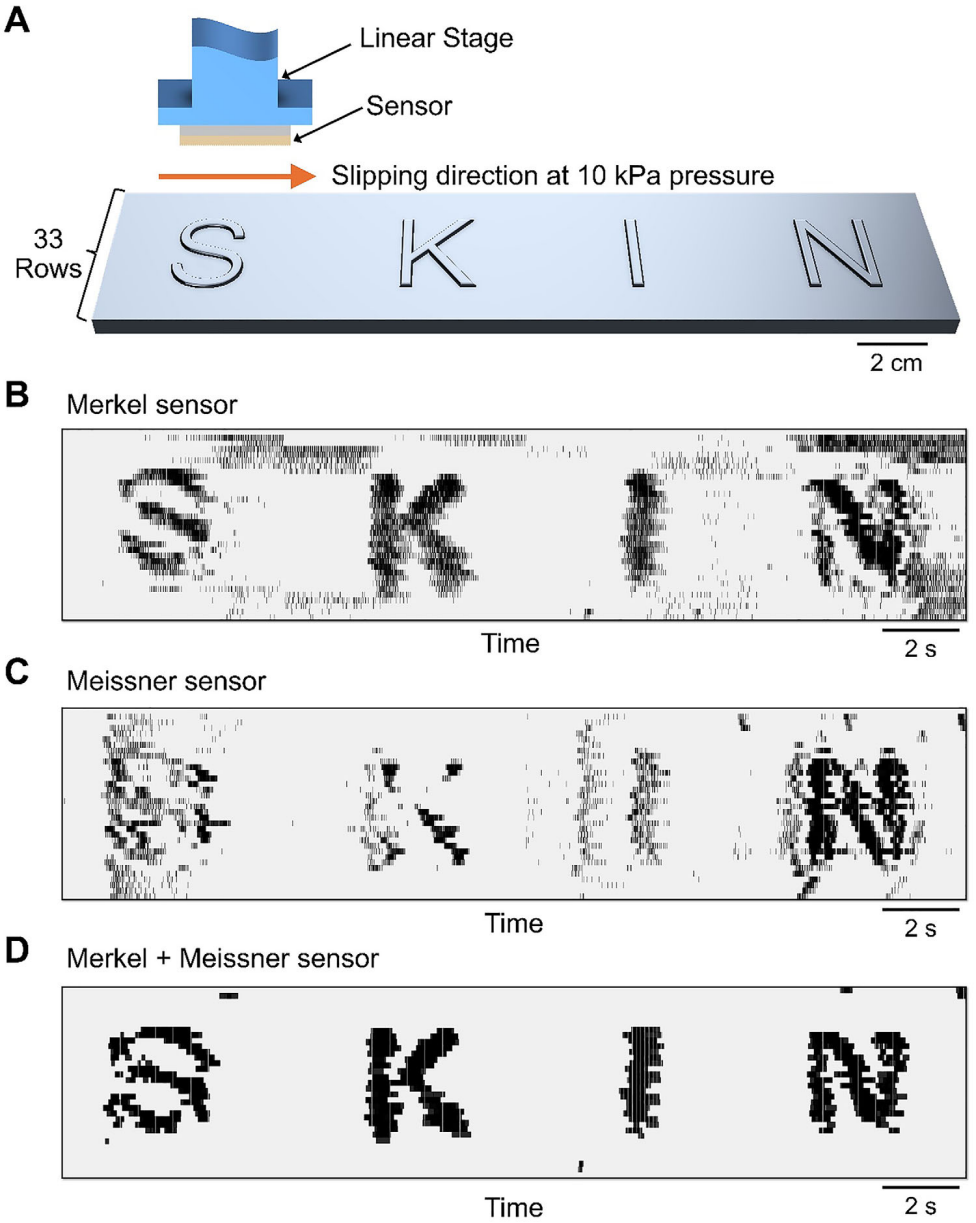
Multimodal tactile regeneration of embossed letters. (A) Schematic of raised “SKIN” molds (≈1 mm embossed height) scanned over our sensor. (B) Regenerated raster plots from Merkel sensor signals. (C) Regenerated raster plots from Meissner sensor signals. (D) Fusion of Merkel and Meissner signals via rate coding and synchronous temporal coding, resulting in a cleaner and more continuous regeneration of the embossed letters.

To compare signal responses with those of biological mechanoreceptors, we visualized the ability of each sensor to recognize embossed alphabet patterns [46, 47, 48]. The embossed letters “S”, “K”, ‘I’, and “N” (1 mm in height) were individually fabricated by 3D printing (Figure S24) and sequentially scanned across the sensor surface. Each scan comprised 33 rows, with each row slid five times iteratively at 10 mm/s to ensure reproducibility (Figure 6A and Video S2). The acquired signals were converted into spike trains using a Leaky Integrating and Firing (LIF) model that mimics neural action potentials, then visualized as spike raster plots for spatiotemporal visualization. Using the Merkel sensor signal only, the regenerated spike raster plot represents the embossed shapes of the letters, projecting a SA signal encoding of MD (Figure 6B). However, spurious spikes also appeared in non‐embossed regions, indicating noise‐like signals. In contrast, the Meissner sensor signal generated local spikes along the edges of the embossed letters, consistent with RA signal of MC (Figure 6C).

We then adopted neural coding strategies inspired by somatosensory processing of the human brain, namely rate coding and synchronous temporal coding [2], to integrate raster plots of Merkel and Meissner sensors. This strategy stems from the mechanism of the brain for integrating afferent signals from multiple mechanoreceptors. Rate coding refers to a framework in which stimulus intensity is represented by the frequency of spikes generated by a single receptor—with stronger stimuli producing higher firing rates. In contrast, synchronous temporal coding refers to a coding strategy in which the relative timing of spikes across multiple receptors conveys the primary information—such that spikes occurring nearly simultaneously are interpreted as a privileged tactile event. Following this principle, we prioritized regions with high spike frequency and simultaneous activation in the graphs obtained from both sensors, while attenuating isolated and temporally inconsistent spikes. This biological merging strategy demonstrated apparent and distinct reproduction of the embossed letters (Figure 6D). To further examine the readability of the fused raster representation, the regenerated raster image was subjected to an auxiliary qualitative readability check using OpenCV‐based preprocessing and Tesseract OCR. This analysis was used only to confirm whether the regenerated tactile pattern retained a machine‐readable alphabetic form. These results are very similar to the cooperative encoding of SA and RA afferents from signals obtained from MC and MD located in the characteristic region within the somatosensory cortex.

## 3 Conclusion

In this study, we created a multimodal tactile sensor with an artificial papillary structure that resembles human skin. This papillary structure, which resembles the biological locations of MC and MD at the epidermal‐dermal junction, was equipped with two different artificial sensors that allowed for the simultaneous detection of both dynamic and static stimuli. Owing to the elastic modulus contrast between the rigid epidermal layer and the soft dermal layer, the papillary structure redistributes externally applied mechanical stimuli and concentrates stress at sensor‐equivalent locations. This stress routing effectively functions as a mechanical filter, selectively enhancing mechanically relevant signals delivered to each sensor. The Meissner sensor responds rapidly to transient stimuli like sliding and microscopic texture, while the Merkel sensor exhibits a sustained response to continuous pressure and indentation, enabling recognition of macroscopic geometric features. By integrating these two complementary sensory pathways, this tactile system extracts both temporal and spatial characteristics of tactile stimuli, closely replicating the cooperative perception mechanism of human mechanoreceptors. Combining multimodal sensor outputs with machine learning analysis demonstrates accurate recognition of surface textures and object shapes. Inspired by the signal merging strategy in human brain, fusion of two distinct sensor signals improved identification accuracy compared to single modal detection. Notably, these results were achieved through a holistic biomimetic design in which the sensor architecture, spatial placement, and signal processing strategy were all deliberately aligned with the structural organization, functional specialization, and neural information encoding of human mechanoreceptors, thereby providing the capability of complex tactile perception. The proposed multimodal sensor system, inspired by papillary structure, not only bridges the gap between biological tactile conversion and artificial sensing but also offers a versatile platform for next‐generation electronic skin, soft robotics, and prosthetic applications.

## 4 Experimental Section/Method

### 4.1 Materials

Poly(vinylidene fluoride–trifluoroethylene) (PVDF‐TrFE) powder was purchased from Piezotech. Polyvinyl chloride, dibutyl adipate (DBA, 99%), and 1‐ethyl‐3‐methylimidazolium bis(trifluoromethylsulfonyl) imide (EMIM‐TFSI, > 99%) were obtained from Sigma–Aldrich. Tetrahydrofuran was used as the solvent for polymer gel preparation and purchased from Sigma–Aldrich. Commercial poly(vinylidene fluoride) (PVDF) films were purchased from Mibago Co. Korea, and used as the piezoelectric layer for the Meissner sensor. Polydimethylsiloxane (PDMS, Sylgard 184, Dow Corning) and EcoFlex 00–10 (Smooth‐On Inc.) were used to construct the epidermal and dermal layers, respectively.

### 4.2 Fabrication of Meissner Sensor

The fabrication process of Meissner sensor is shown in Figure S2. The Meissner sensor operates based on the piezoelectric effect, converting mechanical deformation into electrical signals. Commercial PVDF film was purchased and used as the sensing layer owing to its high piezoelectric coefficient and flexibility. The PVDF film was cut into a cross shape through razor cutting, and both sides were coated with a conductive silver (Ag) layer by sputtering for 3 min to ensure uniform surface conductivity. Carbon tape was attached to both Ag‐coated surfaces to serve as electrodes. Finally, kapton tape was laminated over the outer surface for electrical insulation and mechanical protection.

### 4.3 Fabrication of Merkel Sensor

The fabrication process of Merkel sensor is shown in Figure S3. The Merkel sensor was engineered to mimic the function of MDs, which exhibit slow‐adapting responses under continuous pressure or deformation. PVDF‐TrFE (2 g), PVC (1 g), DBA (2 g), and EMIM‐TFSI (0.1 g) were dissolved in THF (20 mL) and magnetically stirred for 24 h to obtain a homogeneous composite solution. The mixed solution was drop‐cast onto a cylindrical mold (diameter 5 mm, height 1 mm) and dried at room temperature to form a thin film. After complete solvent evaporation, a copper tape electrode was attached to the bottom surface to collect accumulated charges induced by pressure stimuli, while the top surface was directly interfaced with the papillary‐structured PDMS layer. Upon mechanical loading, the PVDF‐TrFE layer generates piezoelectric charges, which induce ionic migration of EMIM‐TFSI within the PVC–DBA matrix. The resulting ion‐assisted polarization prolongs the voltage response during continuous pressure, reproducing the sustained SA response characteristic of biological MDs.

### 4.4 Fabrication of Complete Sensor

The fabrication process of complete sensor is shown in Figure S1. To integrate the Meissner and Merkel sensors within a single structure, the multilayer tactile sensor was fabricated to mimic the epidermis–dermis architecture of human skin, incorporating both papillary and fingerprint structures. A lower mold replicating the geometric features of human fingerprints was fabricated and filled with PDMS (base: curing agent = 5:1). A papillary‐structured upper mold was then placed on top, and the entire mold assembly was maintained under vacuum for 1 h to remove trapped air bubbles. The PDMS layer was cured at 60°C for 24 h to yield an artificial epidermis. The resulting layer featured fingerprint ridges on the top surface and papillae protrusions on the bottom surface, analogous to the epidermal–dermal junction in biological skin. Based on the stress concentration profiles obtained from FEA, the Meissner sensor was placed between the papillary ridges, while the Merkel sensor was embedded directly beneath ridge tips. This configuration allows each sensor to independently respond to lateral motion and normal stress, respectively. Subsequently, the papillary‐patterned epidermal layer with sensors was cast onto a mold filled with EcoFlex 00–10 to form a soft dermal layer. The assembly was held under vacuum for 1 h to ensure complete filling and cured at room temperature for 1 h. The low modulus of EcoFlex provides a mechanical contrast to PDMS, enabling controlled stress transmission across the bilayer interface.

### 4.5 Finite Element Analysis Simulation

Finite element analysis (FEA) was performed using COMSOL Multiphysics 5.3 to investigate the mechanical stress distribution within the papillary‐structured skin model. The geometry was designed based on anatomical references of human dermal–epidermal junctions, consisting of a ridge–valley papillary structure. The epidermal and dermal layers were modeled with heights of 0.4 and 1.0 mm, respectively, and the interdigitated papillary region had a thickness of 0.35 mm. The total model dimensions were 1.75 mm in height and 2.15 mm in width. The Young's modulus of the epidermis and dermis was set to maintain a 4:1 ratio. Both layers were defined as nearly incompressible materials with a Poisson's ratio of 0.48 and a density of 1100 kg m^−^^3^. To ensure consistency between the simulation and experimental validation, the FEA loading condition was revised to match the experimental loading condition of 300 gf, corresponding to 2.94 N. To examine mechanical signal transduction at receptor‐equivalent sites, both normal and lateral forces were applied to the top surface of the epidermis, simulating pressing and sliding stimuli, respectively. The load‐matched simulations in Figure 2, Figure S7, and Figure S13 were performed under 2.94 N, whereas the 0.1 N simulations in Figures S6 and S8 were used to examine geometry‐dependent and load‐scaling trends. The bottom surface of the dermis was fixed in all directions, while the lateral boundaries were free. A fine mesh was employed near the papillary interface to accurately resolve concentrations of local stress. The maximum and average von Mises stresses were extracted at two representative positions corresponding to the MD (beneath ridge tip) and MC (between papillary ridges). This simulation framework allowed quantitative analysis of how papillary geometry modulates the spatial distribution of normal and shear stresses within the skin‐like multilayer, thereby guiding the design of the bioinspired sensor architecture.

### 4.6 Data Collection During Recognition Tests

For all three recognition experiments shown in Figure 5, the experiments were conducted under identical mechanical conditions, with a constant normal force of 300 gf applied to the sensor surface and a lateral sliding speed of 5.00 mm s^−^^1^ along a fixed direction. For each sample, approximately 200 slip trials were performed. During each trial, time‐domain voltage signals were recorded simultaneously from the Merkel and Meissner sensors by connecting them to separate channels of an oscilloscope, enabling fully synchronized acquisition of SA and RA responses to the same sample. The recorded time‐domain signals were converted into spectrogram images using short‐time Fourier transform (STFT) with a frequency window set between 0 and 100 Hz. These STFT maps were used as the input datasets for the CNN classification model and t‐SNE‐based feature visualization. For single‐channel classification, STFT spectrograms obtained from either the Merkel or Meissner sensor were used as the CNN input. For dual‐channel fabric–shape recognition, Merkel and Meissner STFT spectrograms obtained from the same trial were paired using the same class label and file index. The paired spectrograms were then used as two separate inputs to a two‐stream CNN. Each sensor input was processed through an individual convolutional branch, and the extracted Merkel and Meissner feature vectors were merged by feature‐level concatenation before the fully connected layer. The concatenated feature vector was then used for softmax classification into 12 fabric–shape classes. No decision‐level voting or post‐classification averaging was used.

### 4.7 Statistical Analysis

Statistical analyses were performed using device‐level data obtained from five independently fabricated sensors unless otherwise specified. For pressure‐sensing and grating‐slip measurements, each sensor was tested repeatedly under the same condition, and the representative response value for each device was calculated before statistical comparison. Data are presented as mean ± standard deviation (SD), and error bars indicate device‐to‐device variation from five independent sensors. For two‐group comparisons, such as sensors with and without the papillary structure, Welch's two‐sample t‐test was used because it does not assume equal variance between groups. For classification experiments, approximately 200 slip trials were collected per class, and CNN classification accuracy was calculated separately for each independent sensor using the same preprocessing and network architecture. Comparisons among Merkel‐only, Meissner‐only, and combined Merkel–Meissner conditions were analyzed using one‐way ANOVA followed by post hoc pairwise comparisons. A p‐value below 0.05 was considered statistically significant.

### 4.8 Raster Spike Mapping and Fusion of Signals

To regenerate embossed letters, a total of 33 lateral scan rows were tested. Five iterative slip motions were conducted for each row, during which time‐domain voltage signals were recorded from both the Merkel and Meissner sensors. Before spike conversion, the baseline of each voltage signal was corrected by subtracting its mean voltage. The baseline‐corrected signals were then converted into binary spike trains using a leaky integrate‐and‐fire (LIF) neuronal model implemented in Python. The sampling frequency was 1000 Hz for both Merkel and Meissner signals. For the Merkel signal, the LIF threshold and membrane time constant were set to 1.0 × 10^−^^5^ V and 0.1 s, respectively. For the Meissner signal, the LIF threshold and membrane time constant were set to 1.5 × 10^−^^3^ V and 0.003 s, respectively. Channel‐specific LIF thresholds and membrane time constants were used to account for the different signal amplitudes and temporal response characteristics of the Merkel and Meissner sensors, whereas the rate‐coding and synchrony‐coding windows were applied identically to both spike trains after spike conversion. Only positive threshold crossings were counted as spikes.

To fuse Merkel‐ and Meissner‐derived spike rasters, two coding rules were employed: rate coding and synchronous temporal coding. Before fusion, the Meissner spike train was shifted by 200 ms, and the Merkel spike train was resampled to match the length of the Meissner spike train. For rate coding, a merged spike was generated when either the Merkel or Meissner spike count reached at least seven spikes within a 25 ms time window. For synchronous temporal coding, a merged spike was generated when the Merkel and Meissner spike trains exhibited at least five coincident spike bins within a 30 ms time window. The final merged spike value was assigned as one when either the rate‐coding criterion or the synchronous temporal‐coding criterion was satisfied. The resulting fused raster representation is shown in Figure 6D.

To qualitatively evaluate the readability of the fused raster representation, the generated raster image was processed using OpenCV‐based image preprocessing followed by Tesseract OCR in Python. The image was converted to grayscale, binarized using Otsu thresholding, and inverted to obtain a high‐contrast binary image for OCR input. The output was defined as the text string returned by the OCR engine, and this OCR‐based analysis was used only as an auxiliary qualitative readability check, not as a quantitative classification metric.

## Author Contributions

**Seunghwan Seo**: conceptualization, investigation, validation. **Kyoung‐Yong Chun**: investigation, supervision, writing – original draft, writing – review and editing. **Jaehyeong Kim**: investigation, writing – original draft, validation, writing – review and editing. **Chang‐Soo Han**: conceptualization, methodology, investigation, supervision, writing – original draft, writing – review and editing, funding acquisition, project administration. **Bohee Maeng**: methodology, investigation.

## Funding

This work was supported by The Basic Science Research Program (RS‐2023‐00255584) through the National Research Foundation of Korea (NRF) funded by the Ministry of Science and ICT (MSIT) in Korea

## Conflicts of Interest

The authors declare no conflicts of interest.

## Supporting information




**Supporting File 1**: advs76069‐sup‐0001‐SuppMat.docx.

**Supporting File 2**: advs76069‐sup‐0002‐VideoS1.mp4.

**Supporting File 3**: advs76069‐sup‐0003‐VideoS2.mp4

## Data Availability

The data that support the findings of this study are available from the corresponding author upon reasonable request.
