# Supplementary material for: Synergistic Integration of Artificial Merkel Disc and Meissner Corpuscle via Dermal Papillary Structures for Mechanically Filtered Multimodal Tactile Sensing
Source: Adv Sci (Weinh). 2026 Jun 11:e76069. Online ahead of print. doi: 10.1002/advs.76069 (PMC13336984; doi:10.1002/advs.76069)
Supplement: Supplementary file 1 — Supporting File 1: advs76069‐sup‐0001‐SuppMat.docx. [file ADVS-9999-e76069-s002.docx]

Supporting Information

Synergistic Integration of Artificial Merkel Disc and Meissner Corpuscle via Dermal Papillary Structures for Mechanically Filtered Multimodal Tactile Sensing

Jaehyeong Kim^1^, Bohee Maeng^1^, Seunghwan Seo^1^, Kyoung-Yong Chun^2*^ and Chang-Soo Han^1,2*^

Jaehyeong Kim, Bohee Maeng, Seunghwan Seo, Chang-Soo Han

^1^School of Mechanical Engineering, Korea University, Anam-Dong, Seongbuk-Gu, Seoul 02841, Rep. of Korea

Kyoung-Yong Chun, Chang-Soo Han

^2^Center for Somatosensory Molecular-Level Mimicry, Korea University, Anam-Dong, Seongbuk-Gu, Seoul 02841, Rep. of Korea

Table S1. Comparison of representative tactile sensors with the proposed papillary-structure-integrated multimodal tactile sensor.


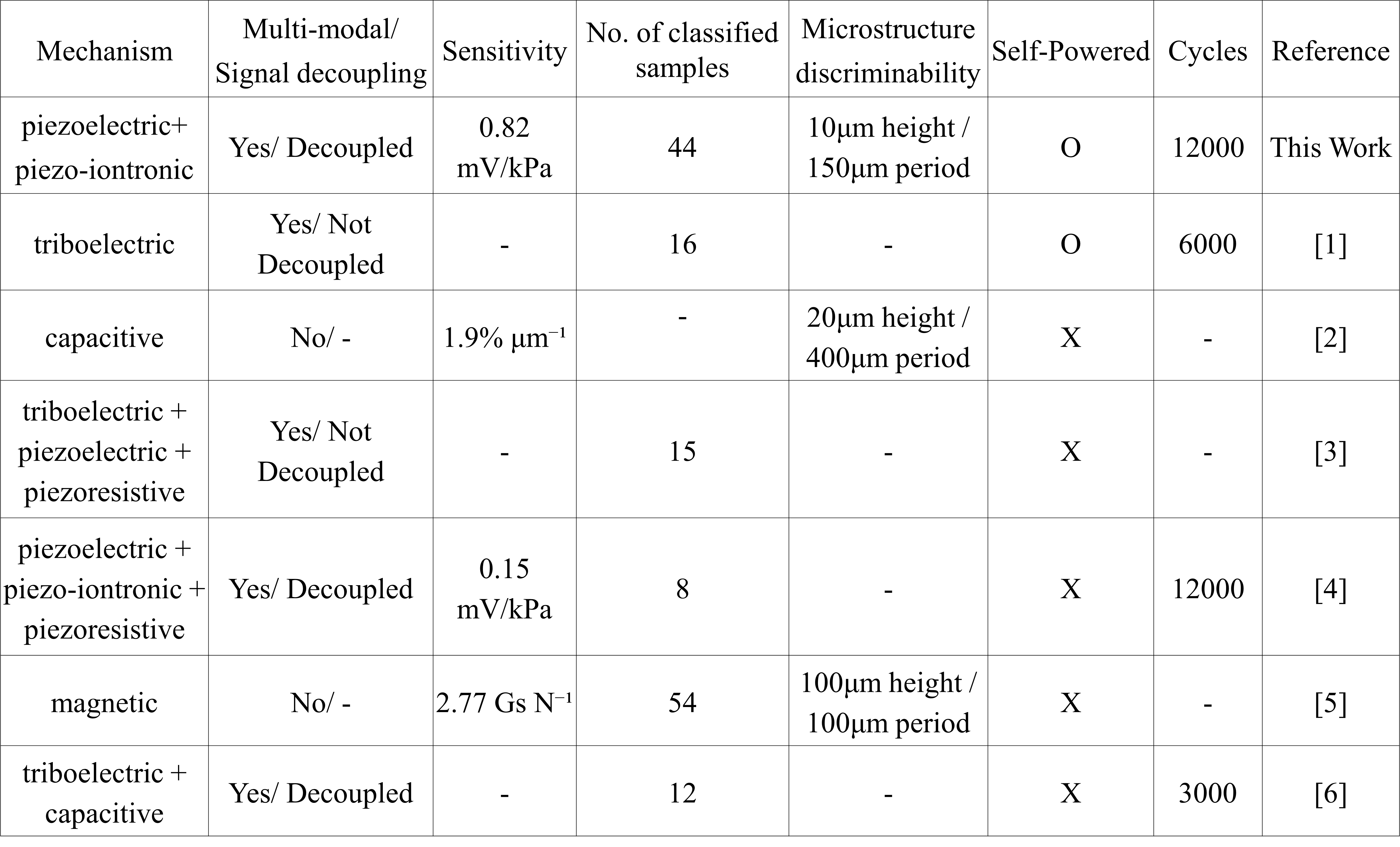


Representative tactile sensors are compared in terms of sensing mechanism, multimodal capability, signal decoupling, sensitivity, classified sample number, microstructure discriminability, fully self-powered operation, cyclic durability, and reference. Here, “fully self-powered” indicates that all sensing channels operate without an external bias voltage. The proposed sensor integrates self-powered piezoelectric Meissner and piezoelectric–iontronic Merkel sensors, enabling structure-guided SA/RA signal decoupling, classification of 44 tactile samples, microstructure discrimination down to 10 μm height and 150 μm period, and stable operation over 12,000 cycles.

Reference

[1] Z. Song, J. Yin, Z. Wang, C. Lu, Z. Yang, Z. Zhao, Z. Lin, J. Wang, C. Wu, J. Cheng, Y. Dai, Y. Zi, S. L. Huang, X. Chen, J. Song, G. Li, W. Ding, *Nano Energy* **2022**, 93.

[2] Y. Wang, J. Zhao, X. Zeng, J. Huang, Y. Wen, J. Brugger, X. Zhang, *Adv. Sci.* **2024**, *11*. 2400479.

[3] G. Lee, J. H. Son, S. Lee, S. W. Kim, D. Kim, N. N. Nguyen, S. G. Lee, K. Cho, *Adv. Sci.* **2021**, *8*. 2002606.

[4] S. Seo, H. M. Na, J. Y. Kim, D. Kim, D. Kim, K. Y. Chun, C. S. Han, *Adv. Funct. Mater.* **2025**, *35*. 2414489.

[5] D. Chen, X. Huang, P. Gao, B. Wang, L. Wei, X. Hu, H. Zeng, J. Liu, A. Song, *IEEE Sens. J.* **2025**, 25, 8175–8186.

[6] Y. Xie, H. Cheng, C. Yuan, L. Zheng, Z. Peng, B. Meng, *Microsyst. Nanoeng.* **2024**, 10, 165.

References in the main text that match references in the Table S1.

[12] Z. Song, J. Yin, Z. Wang, C. Lu, Z. Yang, Z. Zhao, Z. Lin, J. Wang, C. Wu, J. Cheng, Y. Dai, Y. Zi, S. L. Huang, X. Chen, J. Song, G. Li, W. Ding, *Nano Energy* **2022**, 93.

[18] Y. Wang, J. Zhao, X. Zeng, J. Huang, Y. Wen, J. Brugger, X. Zhang, *Adv. Sci.* **2024**, *11*. 2400479.

[24] G. Lee, J. H. Son, S. Lee, S. W. Kim, D. Kim, N. N. Nguyen, S. G. Lee, K. Cho, *Adv. Sci.* **2021**, *8*. 2002606.

[25] S. Seo, H. M. Na, J. Y. Kim, D. Kim, D. Kim, K. Y. Chun, C. S. Han, *Adv. Funct. Mater.* **2025**, *35*. 2414489.

[28] D. Chen, X. Huang, P. Gao, B. Wang, L. Wei, X. Hu, H. Zeng, J. Liu, A. Song, *IEEE Sens. J.* **2025**, 25, 8175–8186.


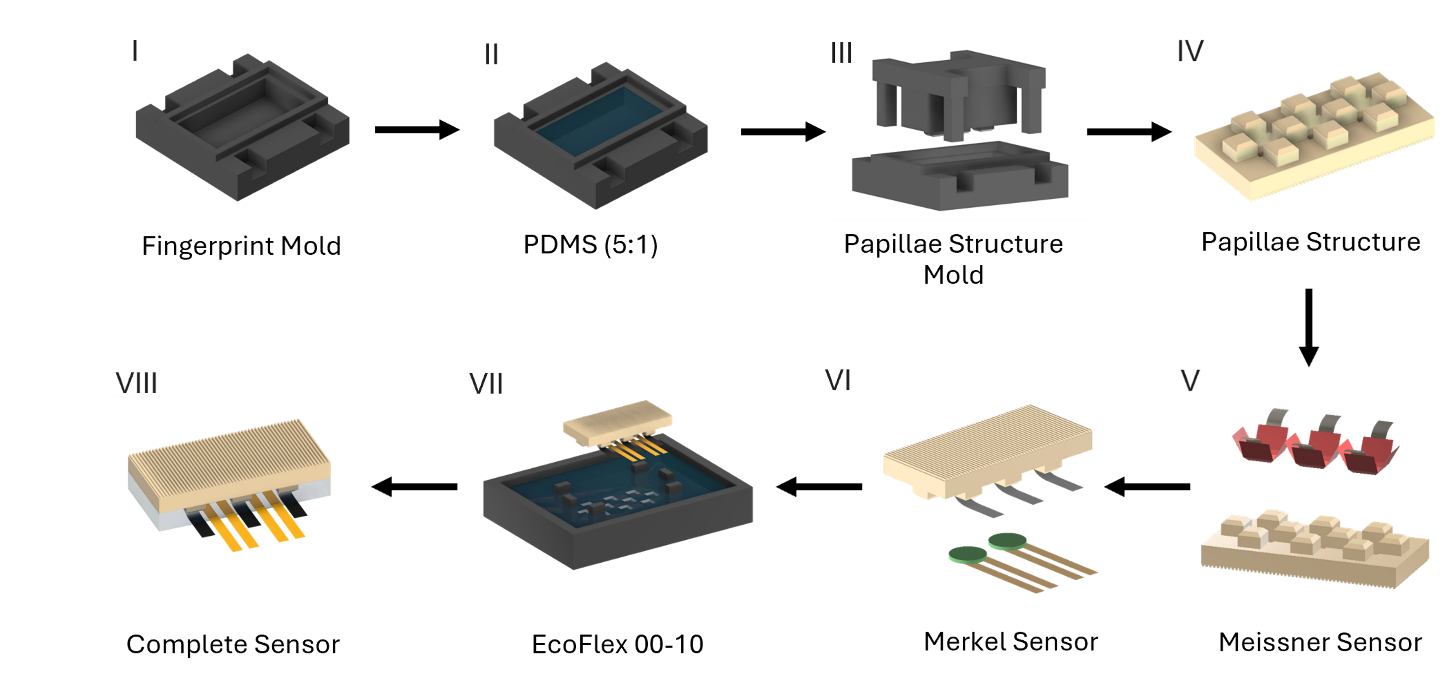


**Figure S1.** Fabrication process of integrating Meissner and Merkel sensors into the artificial papillary structure. Assembly of the complete sensor: PDMS (base:curing agent = 5:1) was poured onto a mold with fingerprint micro-ridges, and a second mold with papillary structures was placed on top. After vacuum degassing for 1 h and curing at 60 °C for 24 h, an epidermal PDMS layer with both fingerprint and papillary structures was obtained. The Meissner sensor was inserted between adjacent papillary ridges, and the Merkel sensor was attached beneath the ridge tips. Finally, Ecoflex 00-10 was cast and cured to form the artificial dermis, completing the integrated dual-mode tactile sensor.


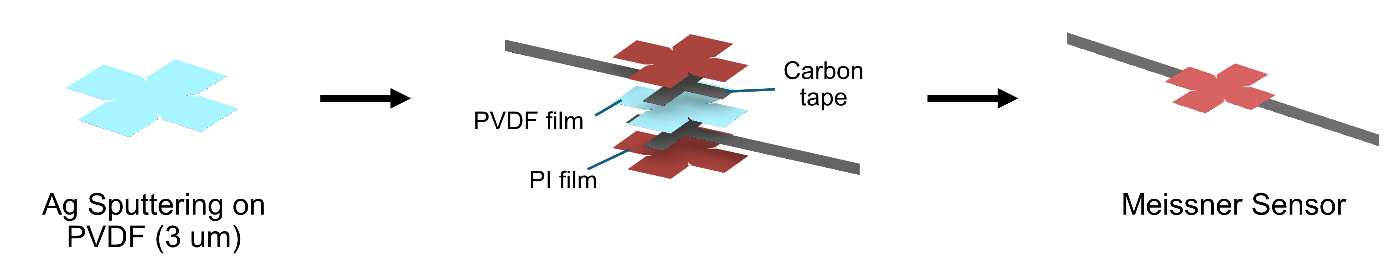


**Figure S2.** Fabrication of the piezoelectric Meissner sensor. A cross-shaped PVDF film was prepared and carbon tape was attached as the top and bottom electrodes, followed by encapsulation with kapton tape to complete the flexible self-powered sensor.


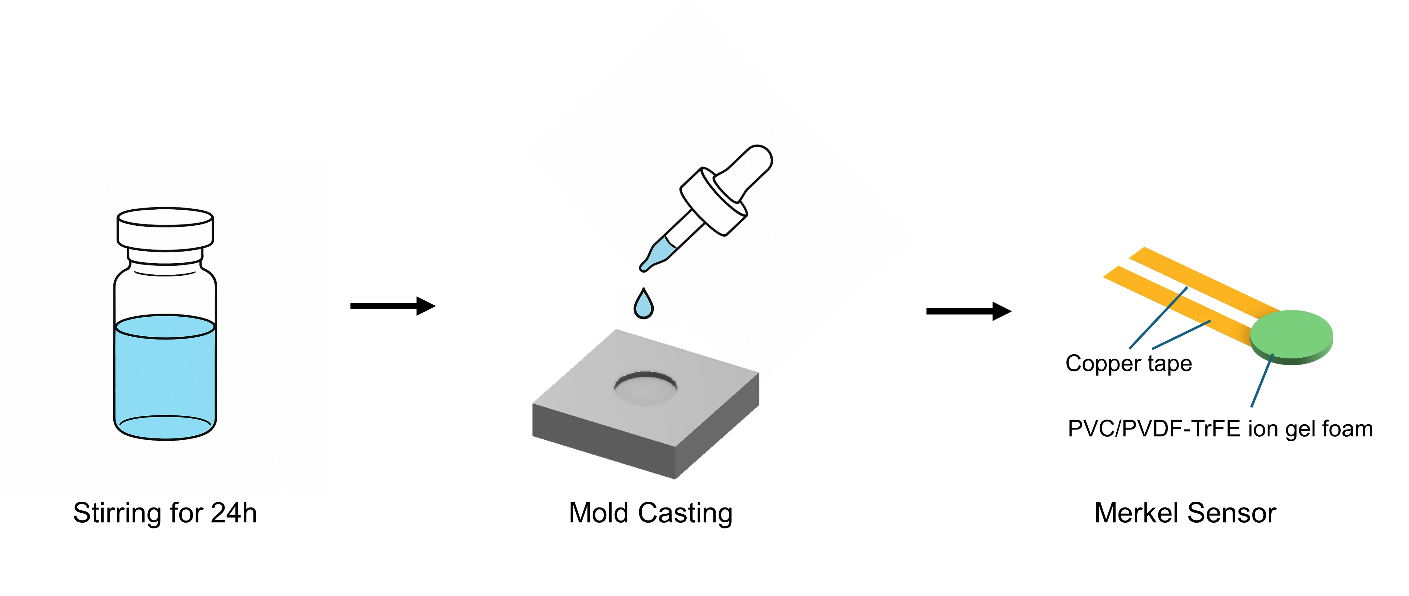


**Figure S3.** Fabrication of the Merkel sensor. PVDF-TrFE, PVC, DBA, and EMIM-TFSI were dissolved in THF and magnetically stirred for 24 h to form a homogeneous solution, which was then cast into a mold and solidified to obtain an ion gel foam film; Cu-tape electrodes were attached on both sides to complete the Merkel sensor.


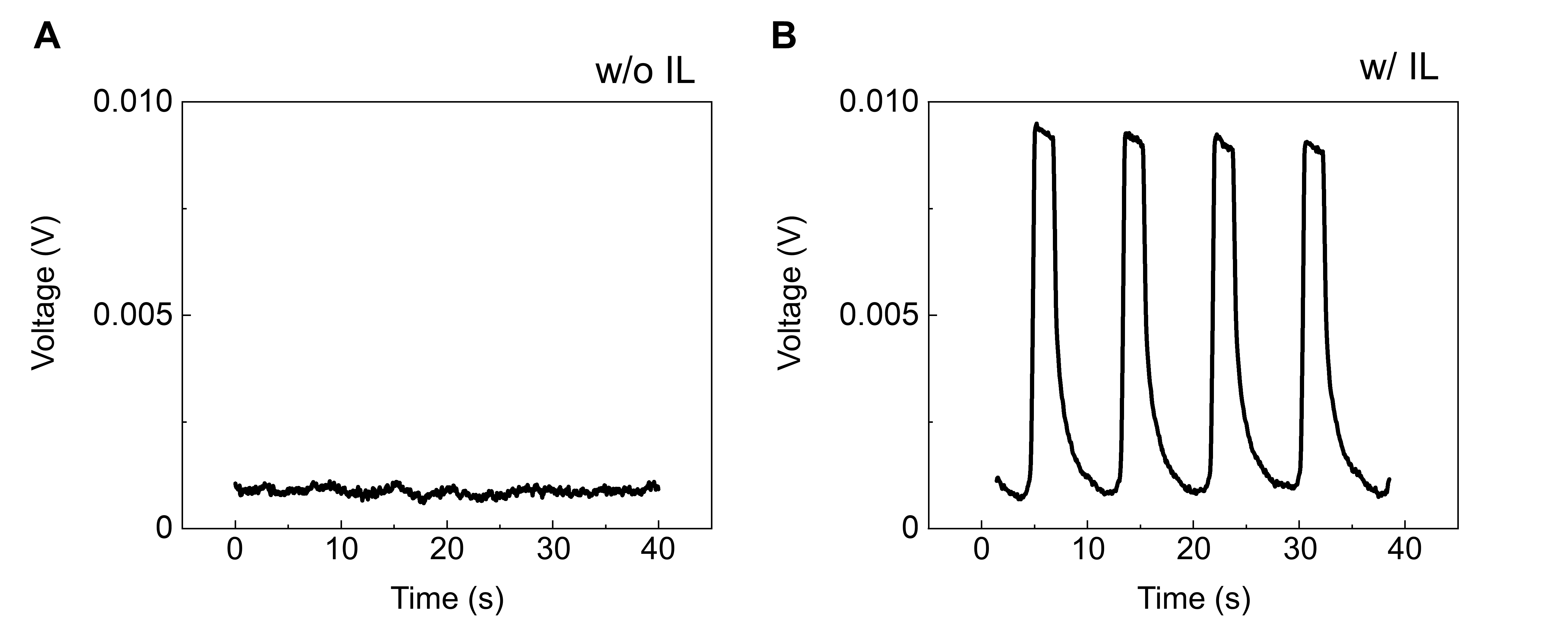


**Figure S4.** Effect of EMIM-TFSI on the sustained SA signal response of the Merkel sensor. The Merkel sensor used in this study was implemented based on the PVDF-TrFE/PVC/DBA/EMIM-TFSI piezo-ionogel pressure sensor previously reported by Maeng et al. ^[35]^. Voltage responses were compared between the ionic-liquid-free control sensor, composed of the same PVDF-TrFE/PVC/DBA matrix without EMIM-TFSI, and the EMIM-TFSI-containing Merkel sensor under repeated static loading of 20 gf, corresponding to approximately 10 kPa. The IL-free control showed only noise-level output without a distinguishable pressure-induced response, whereas the EMIM-TFSI-containing Merkel sensor exhibited clear and sustained voltage responses during the holding period. This comparison demonstrates that EMIM-TFSI-mediated ionic migration is essential for generating the SA signal of the Merkel sensor.

**
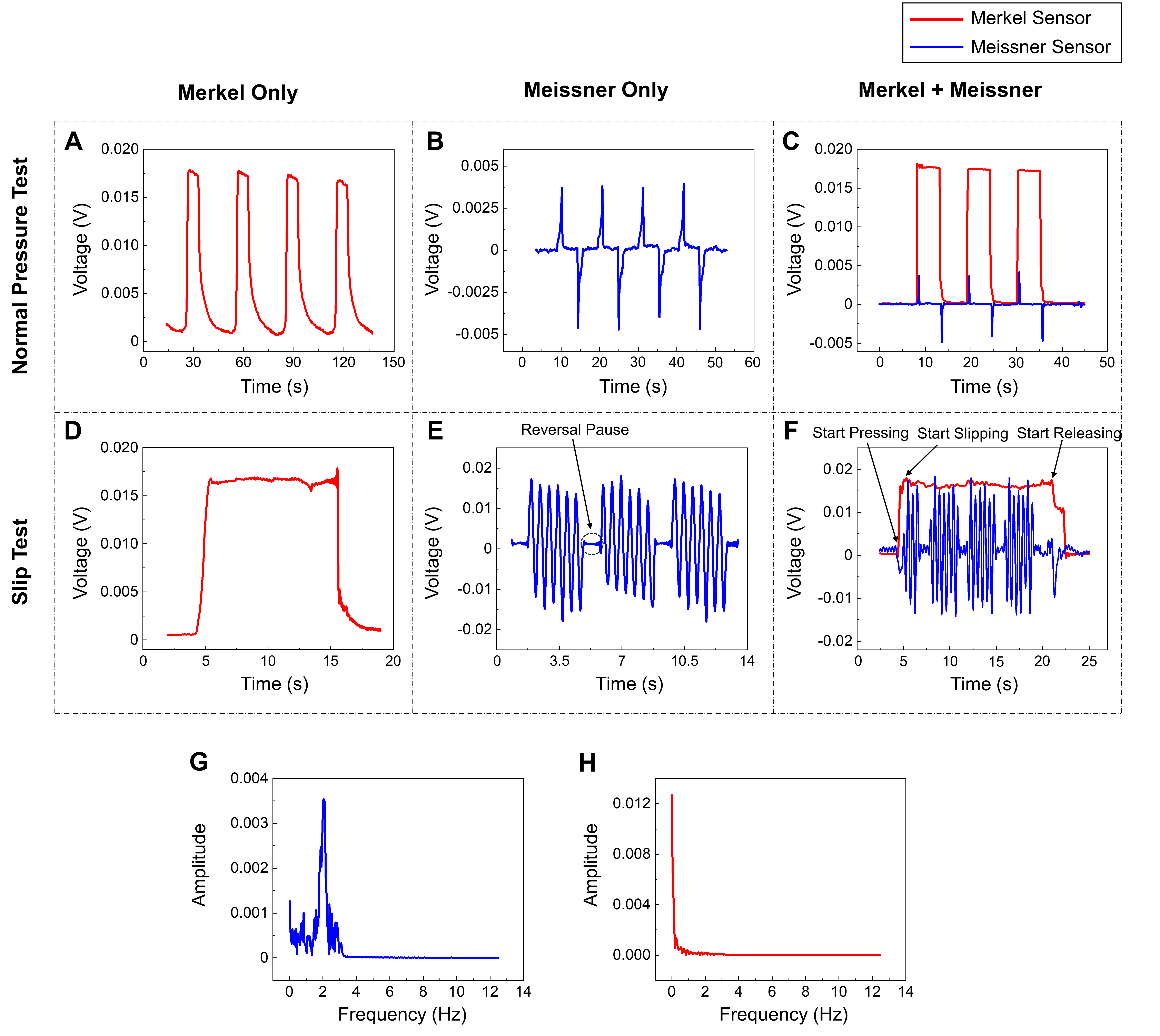
**

**Figure S5.** Evaluation of signal crosstalk between Merkel and Meissner sensors under normal pressure and slip conditions. (A–C) Voltage responses under repeated normal pressure of 200 gf for three configurations: (A) papillary structure with only the Merkel sensor, (B) papillary structure with only the Meissner sensor, and (C) papillary structure with both Merkel and Meissner sensors. The Merkel sensor shows a sustained pressure response during the holding period, whereas the Meissner sensor responds only to transient loading and unloading events. In the dual-sensor configuration, these distinct response characteristics are maintained. (D–F) Voltage responses under slip testing using a grating with a 5 mm period pressed onto the sensor with a normal load of 200 gf and slid back and forth at 10 mm s⁻¹, corresponding to an imposed slip frequency of 2 Hz. (D) The Merkel sensor responds to the normal preload but does not clearly encode the slip-induced periodic vibration. (E) The Meissner sensor generates periodic responses during slip and returns to baseline during the reversal pause, where lateral motion is temporarily stopped while the normal preload is maintained. (F) In the dual-sensor configuration, the Merkel channel preserves the sustained pressure envelope during pressing, slipping, reversal pause, and releasing, whereas the Meissner channel selectively detects the dynamic slip component and returns to baseline during the reversal pause.

(G,H) FFT spectra extracted from the Meissner (G) and Merkel (H) channels in the dual-sensor slip test. The Meissner channel exhibits a distinct peak at the imposed slip frequency of 2 Hz, whereas the Merkel channel does not show a discernible 2 Hz peak. These results confirm that the Merkel and Meissner sensors preferentially encode sustained normal pressure and slip-induced dynamic vibration, respectively, under combined loading conditions.


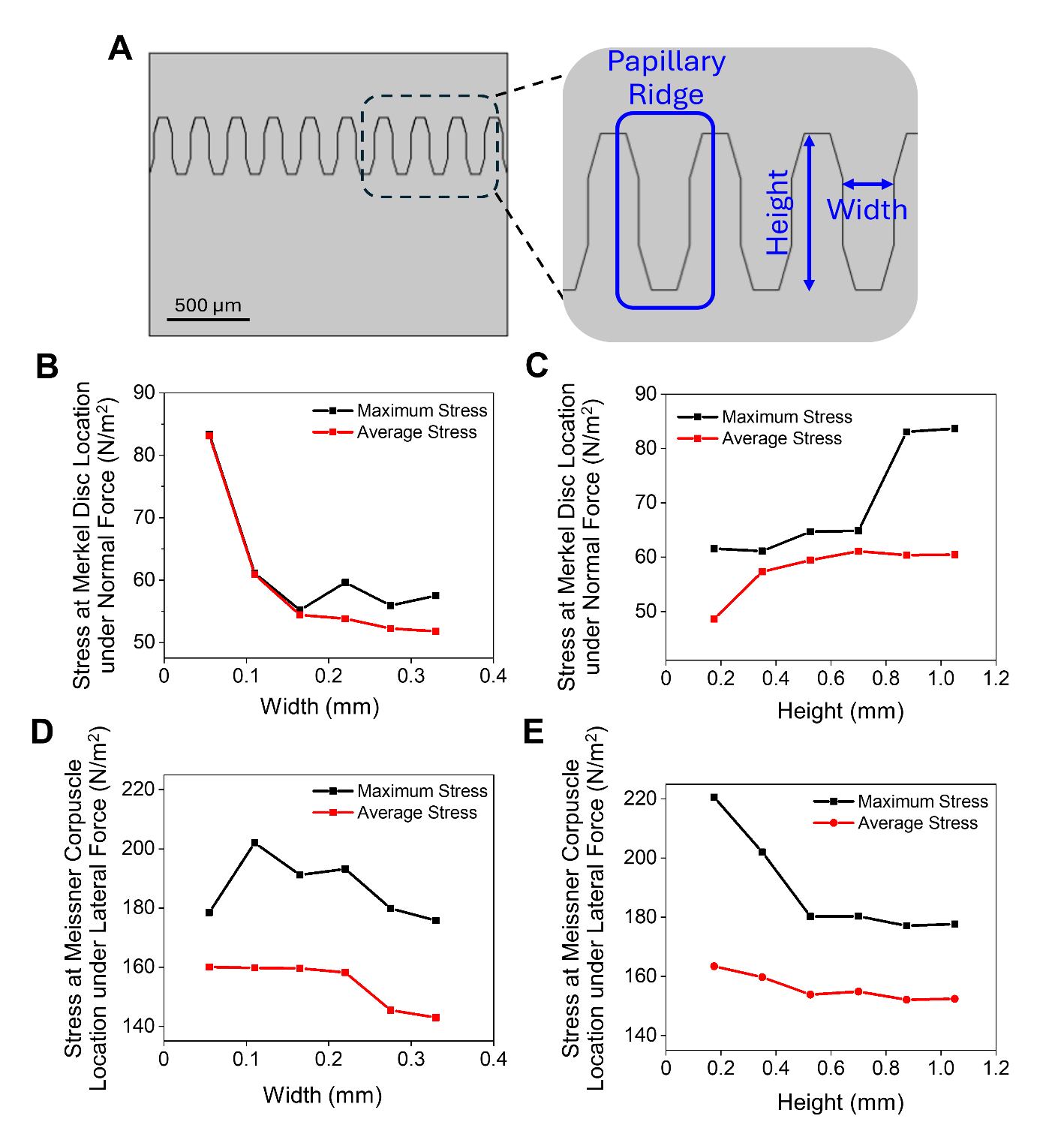


**Figure S6.** Finite element analysis of geometric parameters of papillary ridge affecting stress concentration in the papillary structure. (A) Geometry of the modeled papillary structure in human skin, where the epidermal papillary ridges protrude downward toward the dermis layer. The variation in papillary ridge geometry determines how stress is distributed between the epidermis and dermis layers. (B) Simulated maximum and average stress values at the Merkel disc location under a normal load (0.1 N) as a function of ridge width. Wider ridges lead to smaller stress concentration beneath the ridge tip. (C) Simulated maximum and average stress values at the Merkel disc location under a normal load (0.1 N) as a function of ridge height. Increasing ridge height enhances stress concentration below the ridge tip. (D) Simulated maximum and average stress values at the Meissner corpuscle location under a lateral load (0.1 N) as a function of ridge width. The stress decreases as the ridge width increases. (E) Simulated maximum and average stress values at the Meissner corpuscle location under a lateral load (0.1 N) as a function of ridge height. Higher ridge height reduces stress in the valley region.


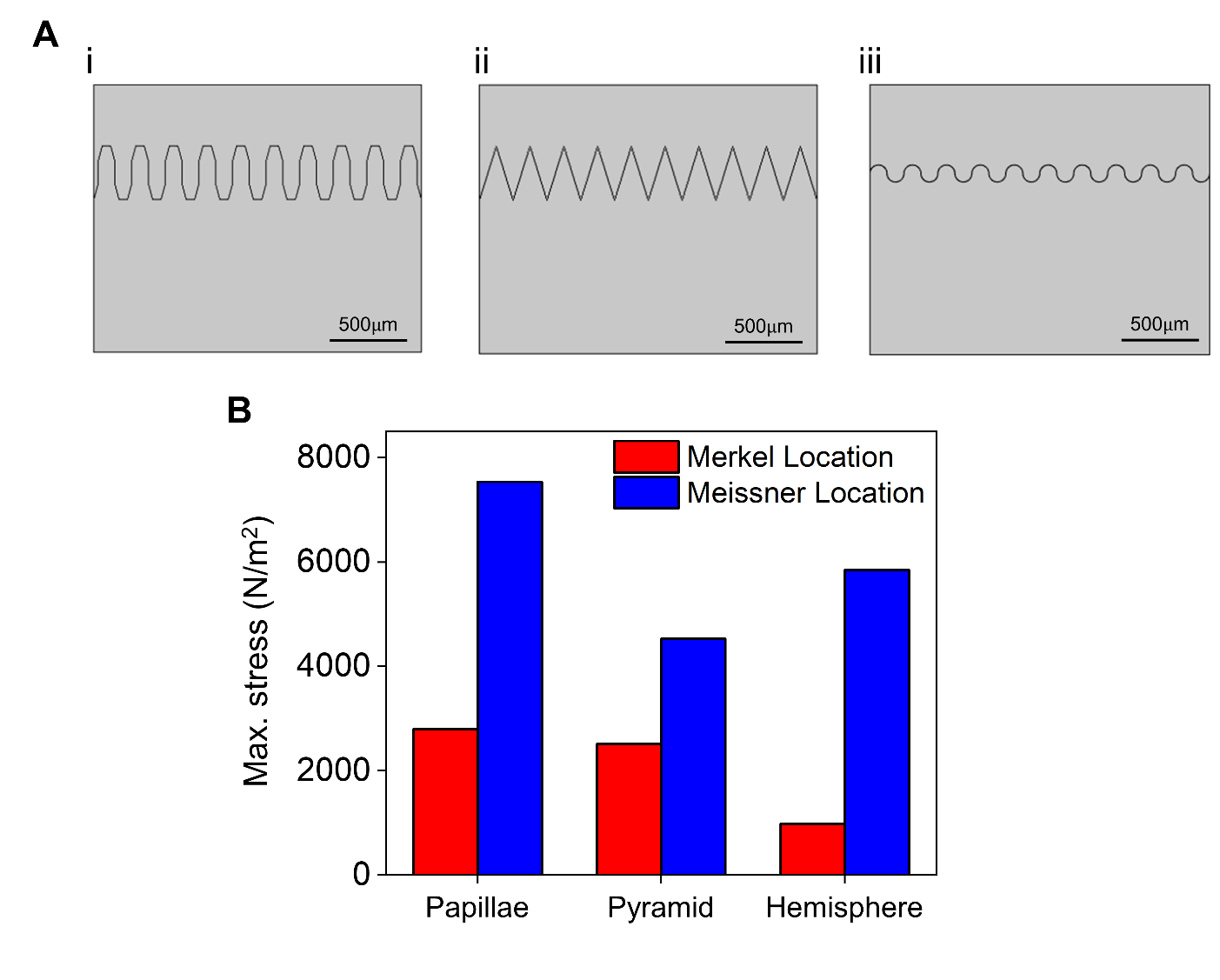


**Figure S7.** Comparative finite element analysis of papillary, pyramidal, and hemispherical microstructures. (A) Cross-sectional geometries of the three microstructures used for FEA: (i) papillary, (ii) pyramidal, and (iii) hemispherical structures. All structures were modeled with identical material properties and comparable geometric footprints. (B) Maximum stress values extracted at the Merkel-disc-equivalent location under a normal force of 2.94 N and at the Meissner-corpuscle-equivalent location under a lateral force of 2.94 N. The papillary structure exhibited the highest stress concentration at both receptor-equivalent locations, indicating its functional advantage for mechanically enhancing both static Merkel sensing and dynamic Meissner sensing pathways.


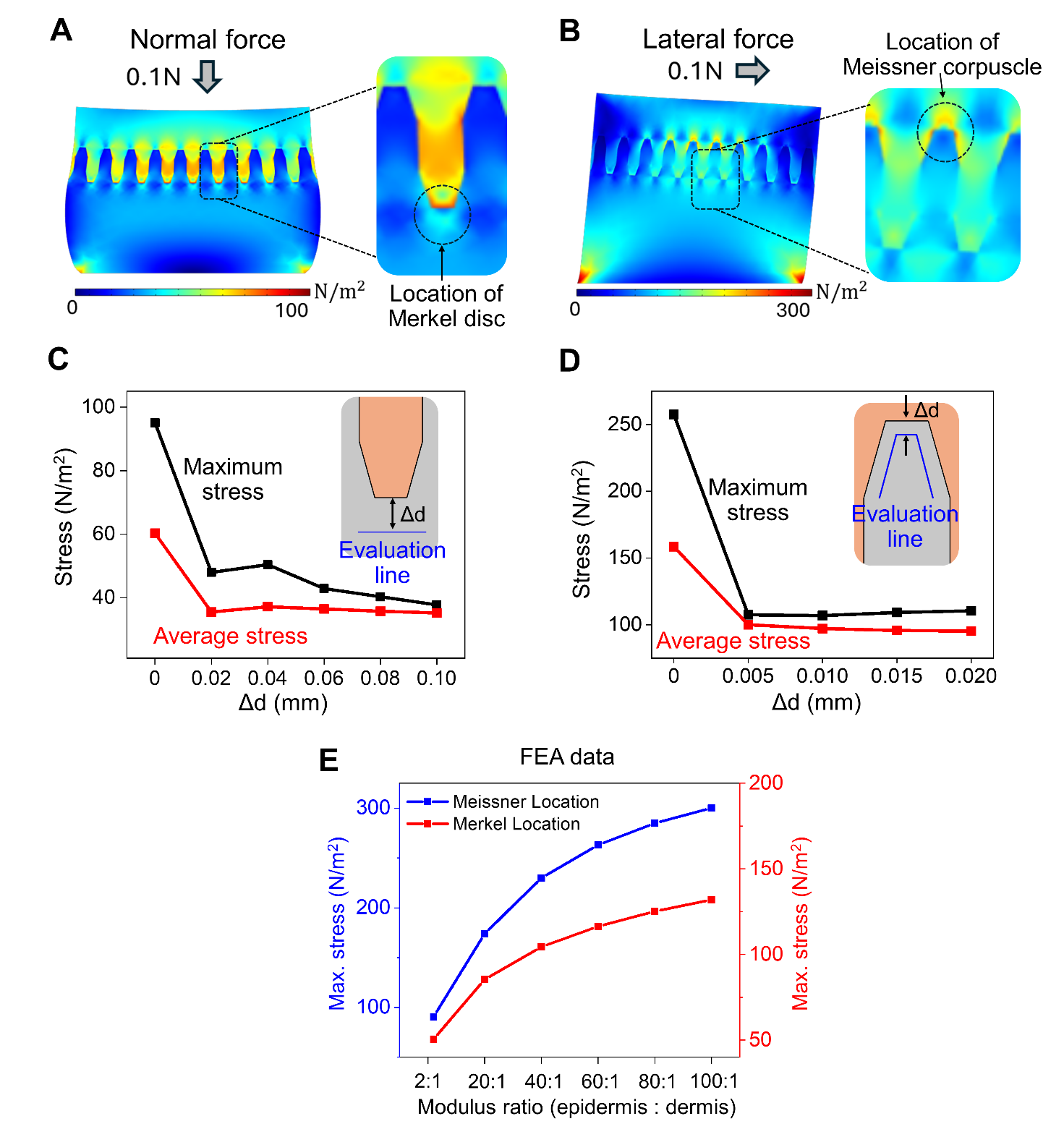


**Figure S8.** Finite element analysis under a low-load condition of 0.1 N. (A) Stress distribution in the papillary structure under a normal force of 0.1 N, showing localized stress concentration beneath the papillary ridge tip, corresponding to the Merkel disc location. (B) Stress distribution under a lateral force of 0.1 N, showing stress concentration in the valley region between adjacent papillary ridges, corresponding to the Meissner corpuscle location. (C) Average and maximum stress profiles extracted along a virtual evaluation line placed at a vertical distance Δd from the papillary ridge tip under normal loading. The maximum stress was highest at Δd = 0 mm, indicating that the region directly beneath the papillary ridge tip is the mechanically dominant location for the Merkel sensor. (D) Average and maximum stress profiles extracted along a virtual evaluation line placed at a horizontal distance Δd from the valley region under lateral loading. The maximum stress was highest at Δd = 0 mm, confirming that the valley region between adjacent papillary ridges is the mechanically dominant location for the Meissner sensor. (E) Maximum stress at the functionally equivalent locations of the Merkel disc under normal force and the Meissner corpuscle under lateral force as a function of the epidermis-to-dermis modulus ratio. These results confirm that the qualitative stress-localization pattern observed under the 0.1 N loading condition is consistent with the load-matched 2.94 N simulation in Figure 2, indicating that load scaling changes the absolute stress magnitude but does not alter the spatial stress-routing behavior of the papillary structure.


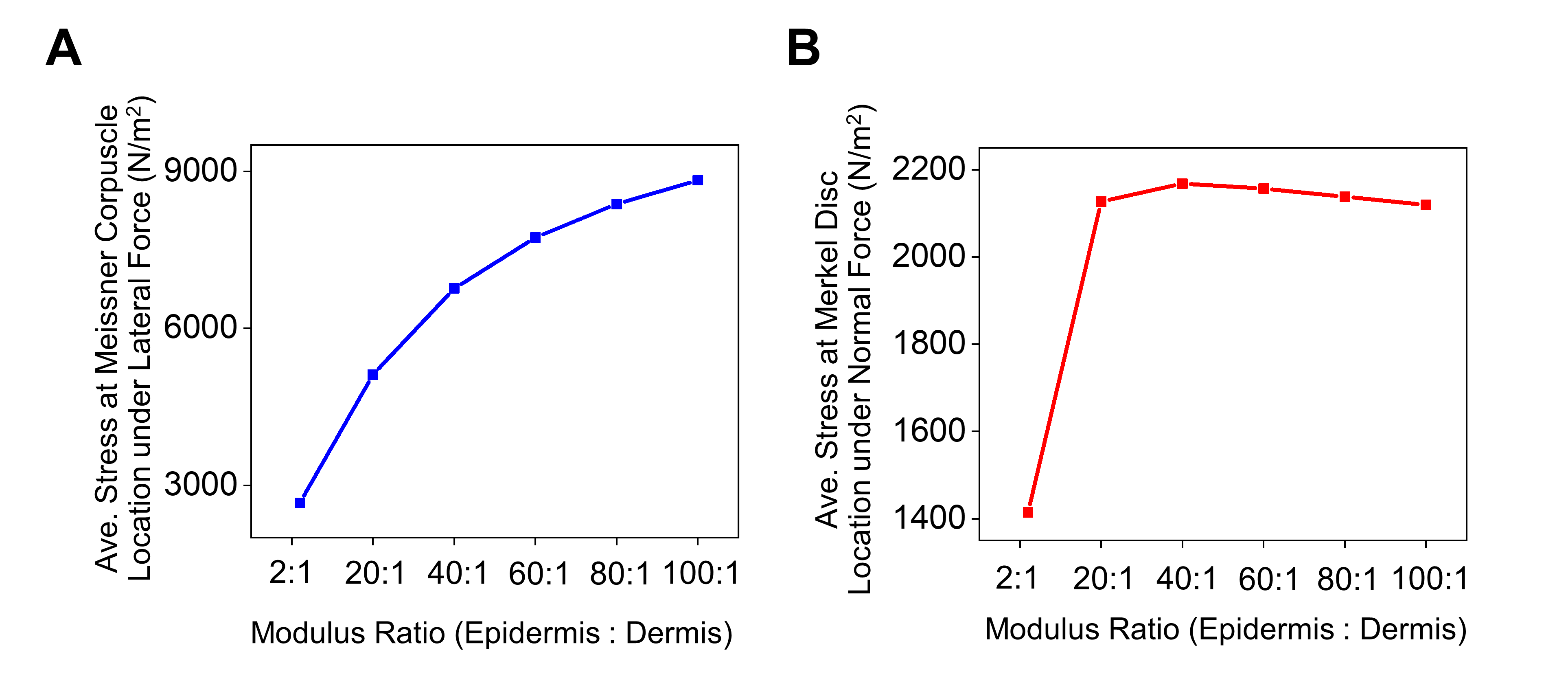


**Figure S9.** Average stresses obtained from FEA at the Merkel disc and Meissner corpuscle locations under normal and lateral loading, respectively, as a function of the epidermis-to-dermis modulus ratio. (A) Average stress at the Meissner corpuscle location under lateral force increases as a function of the modulus ratio between epidermis and dermis. (B) Average stress at the Merkel disc location under normal force increases as a function of the modulus ratio between epidermis and dermis.


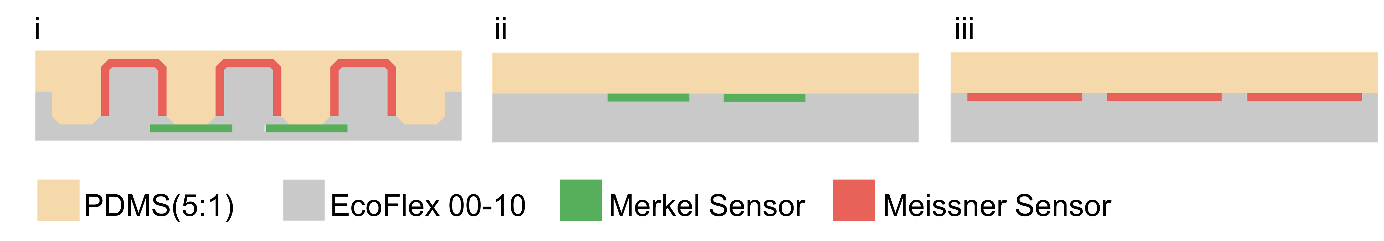


**Figure S10.** Schematic clarification of papillae-integrated and planar control architectures.

(i) Architecture with the papillary structure containing both Merkel and Meissner sensors. The Merkel sensors are positioned beneath the papillary ridge tips, whereas the Meissner sensors are placed between adjacent papillary ridges. (ii) Planar architecture without the papillary structure containing Merkel sensors. (iii) Planar architecture without the papillary structure containing Meissner sensors. The planar architectures were separately prepared for Merkel and Meissner sensing because simultaneous integration of both sensor types within the same planar footprint was not feasible while preserving identical sensor dimensions and spatial separation. For the comparisons in Figure 3, the Merkel sensor response was compared under normal loading, and the Meissner sensor response was compared under dynamic grating-slip loading. All non-geometric variables, including sensor material, sensor dimensions, layer materials, overall footprint, and testing conditions, were kept identical; only the presence or absence of the papillary structure was varied.


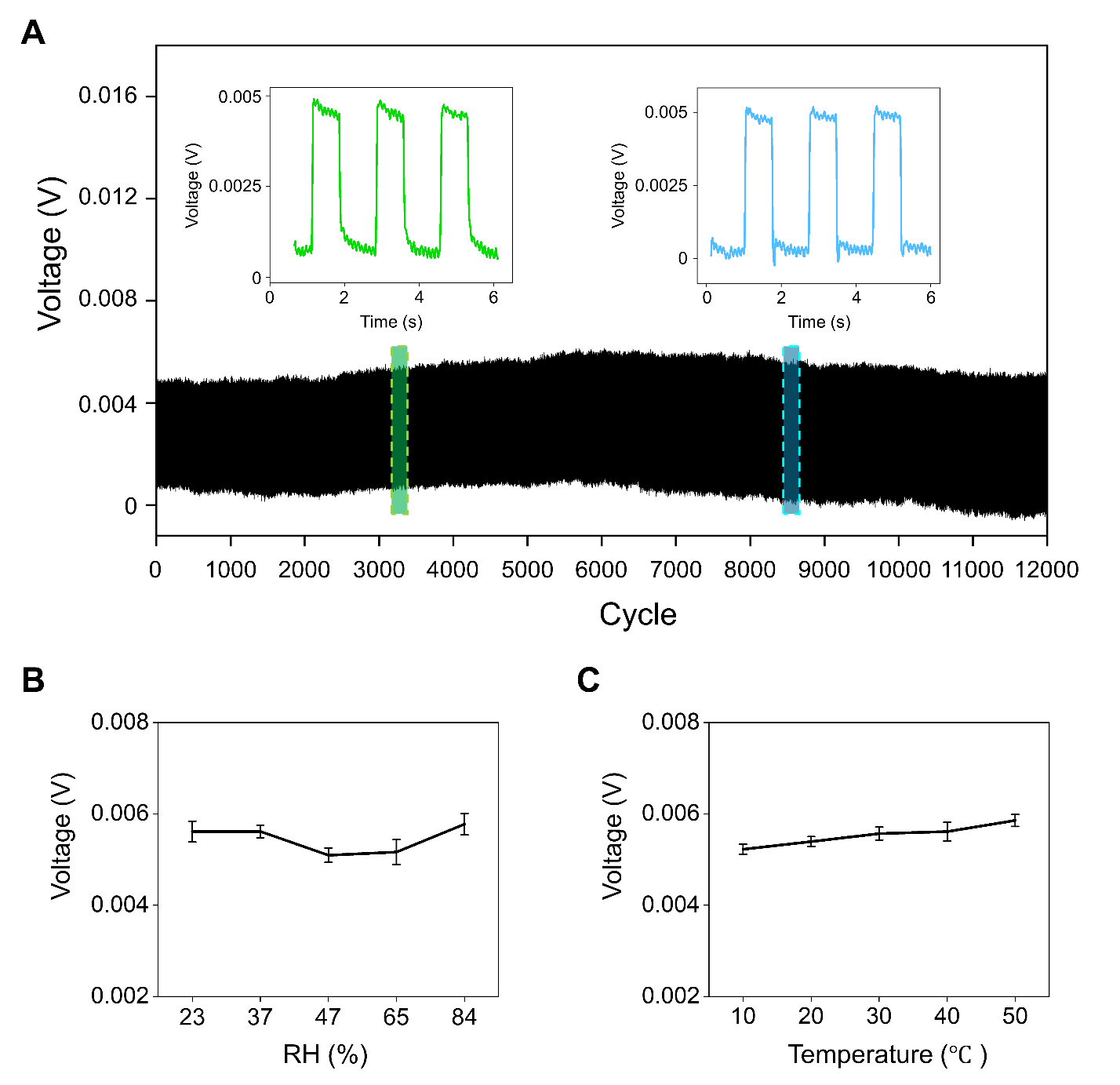


**Figure S11.** Cyclic durability and environmental stability of the Merkel sensor. (A) Long-term cyclic response of the Merkel sensor under repeated loading of 15 gf for 12,000 cycles. Insets show representative voltage responses extracted from early and later cycles, confirming comparable pressure-induced signal shapes during repeated operation. (B) Output voltage of the Merkel sensor measured under different relative humidity conditions from 23% to 84% RH under an applied force of 15 gf. Although slight variations were observed, no clear monotonic RH-dependent trend appeared. (C) Output voltage of the Merkel sensor measured at different temperatures from 10 to 50 °C under an applied force of 15 gf. The output voltage slightly increased with temperature, but no abrupt thermal drift or failure was observed.


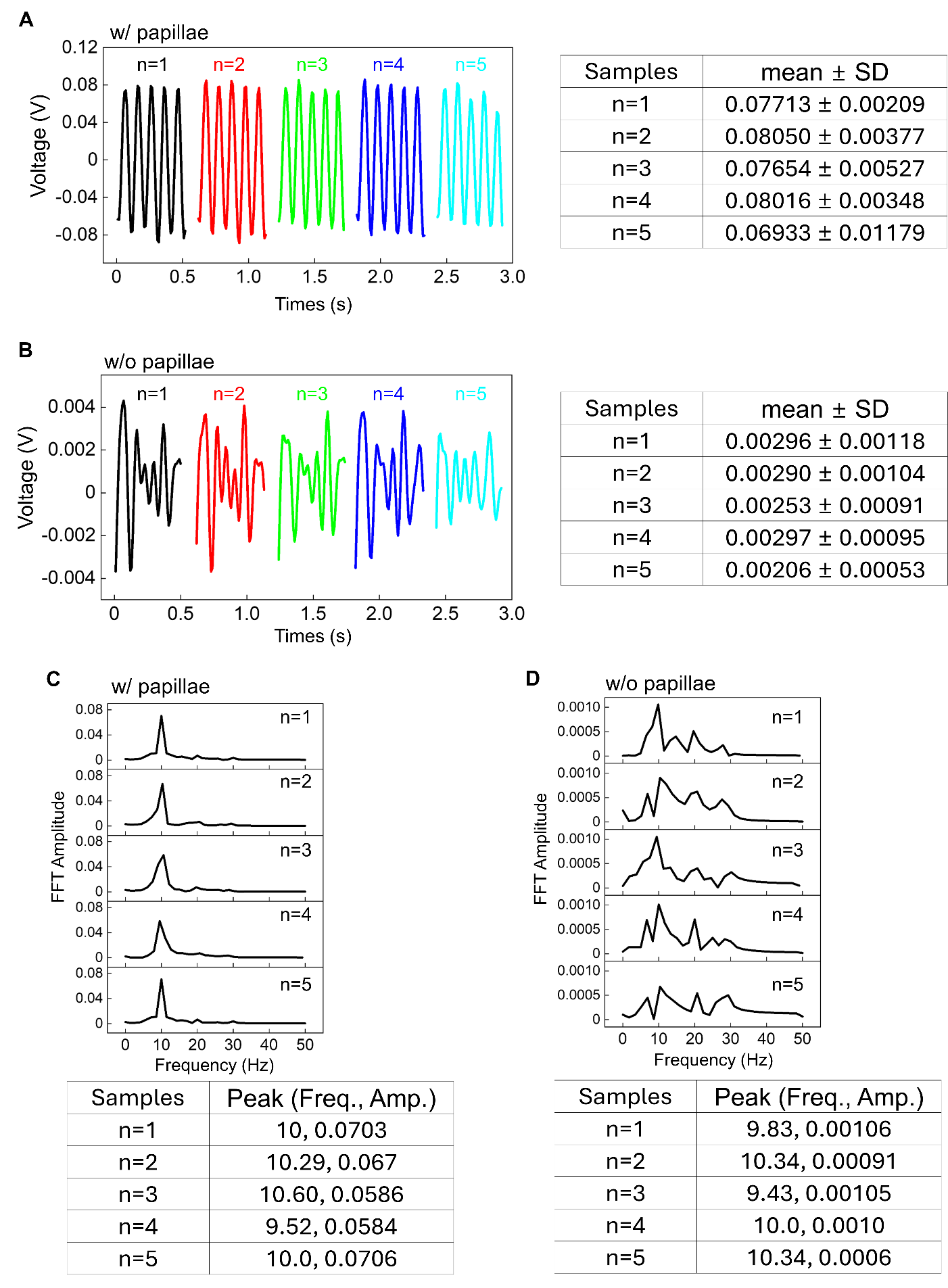


**Figure S12.** Inter-device reproducibility of Meissner sensor responses under 10 Hz grating-slip stimulation. (A and B) Time-domain voltage responses of five independently fabricated Meissner sensors under 50 mm s⁻¹ grating-slip stimulation, corresponding to an imposed frequency of 10 Hz, for architectures (A) with and (B) without the papillary structure. The values in the tables represent the mean peak amplitude ± SD calculated for each device.

(C and D) FFT spectra obtained from the corresponding time-domain signals for architectures (C) with and (D) without the papillary structure. The peak frequency and peak amplitude extracted from each device are summarized below each FFT plot. The architecture with the papillary structure exhibited pronounced spectral peaks near the imposed 10 Hz frequency, whereas the planar architecture without the papillary structure showed weaker and more irregular spectral responses.

**
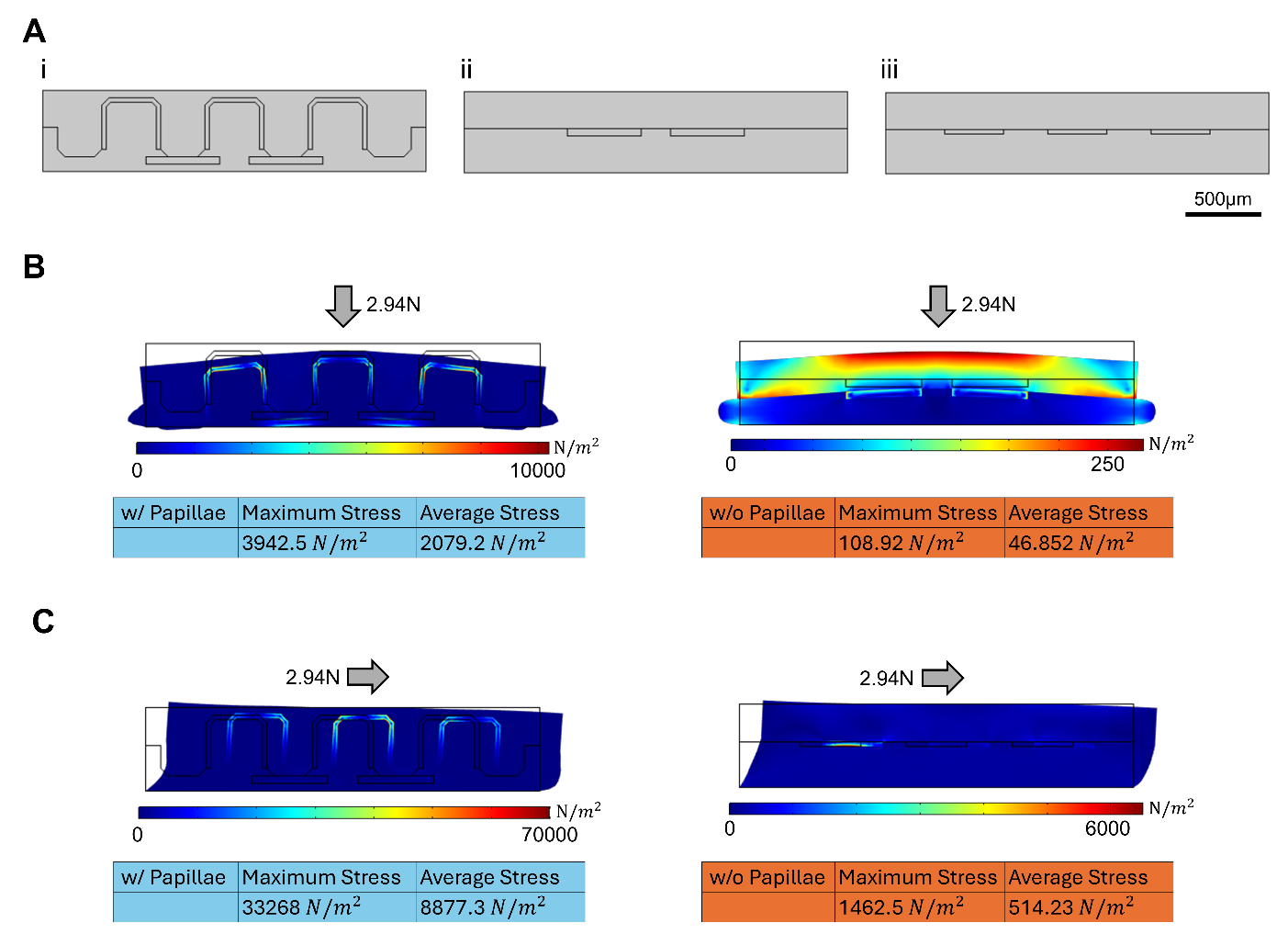
**

**Figure S13.** Finite element analysis of stress distribution in sensor architectures with and without papillae. (A) Structural configurations used for simulation. (i) Proposed sensor architecture with papillary structure containing both Merkel and Meissner sensors. (ii) Planar architecture without papillary structure containing only the Merkel sensor between the artificial dermis and epidermis. (iii) Planar architecture without papillary structure containing only the Meissner sensor between the artificial dermis and epidermis. The papillary geometry enables denser spatial integration of both sensors within the same footprint. In contrast, a planar structure would require a larger area to accommodate the same number of Merkel and Meissner sensors, as illustrated in the schematics. (B) Simulated stress distribution under a normal force of 2.94 N, comparing architectures with and without papillae. The structure with papillae induces substantially higher maximum and average stresses on the Merkel sensor (3942.5 N m⁻² and 2079.2 N m⁻², respectively) compared to the planar structure without papillae (108.92 N m⁻² and 46.852 N m⁻²), indicating enhanced stress concentration under vertical loading. (C) Simulated stress distribution under a lateral force of 2.94 N, evaluating stress transfer to the Meissner sensor. The structure with papillae generates significantly higher maximum and average stresses on the Meissner sensor (33268 N m⁻² and 8877.3 N m⁻², respectively) than the planar structure without papillae (1462.5 N m⁻² and 514.23 N m⁻²), demonstrating improved mechanical coupling under shear loading. Overall, the papillary structure enhances mechanical stress concentration on both sensors under normal and lateral forces, supporting the experimentally observed improvements in static and dynamic sensing performance.


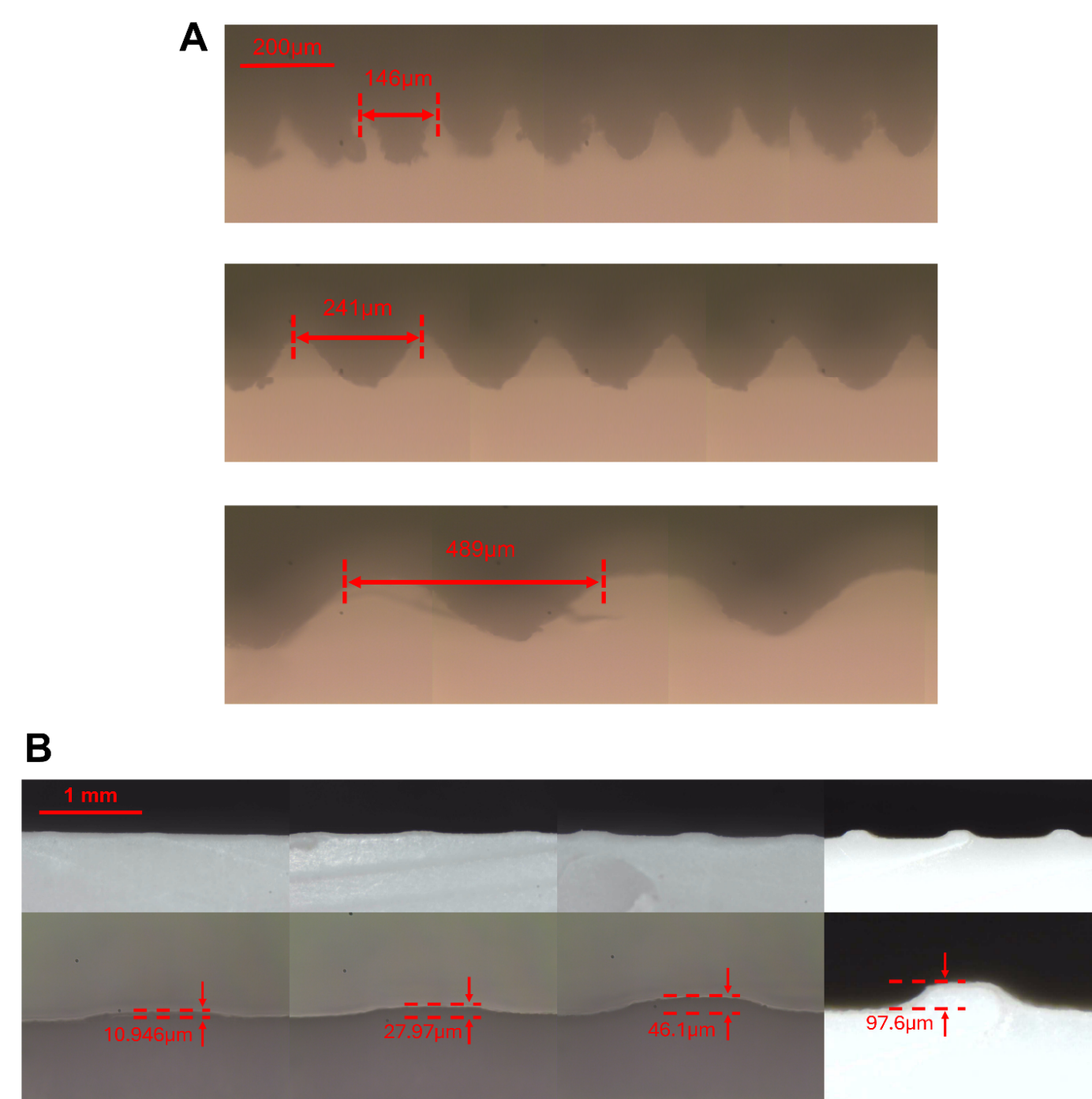


**Figure S14.** Photographs of microscale grating structures showing geometric dimensions. (A) Optical images of the fabricated grating samples with heights of 10, 30, 50, and 100 μm. (B) Optical images of gratings with different spatial periods of 500, 250, and 150 μm used for the experiments.


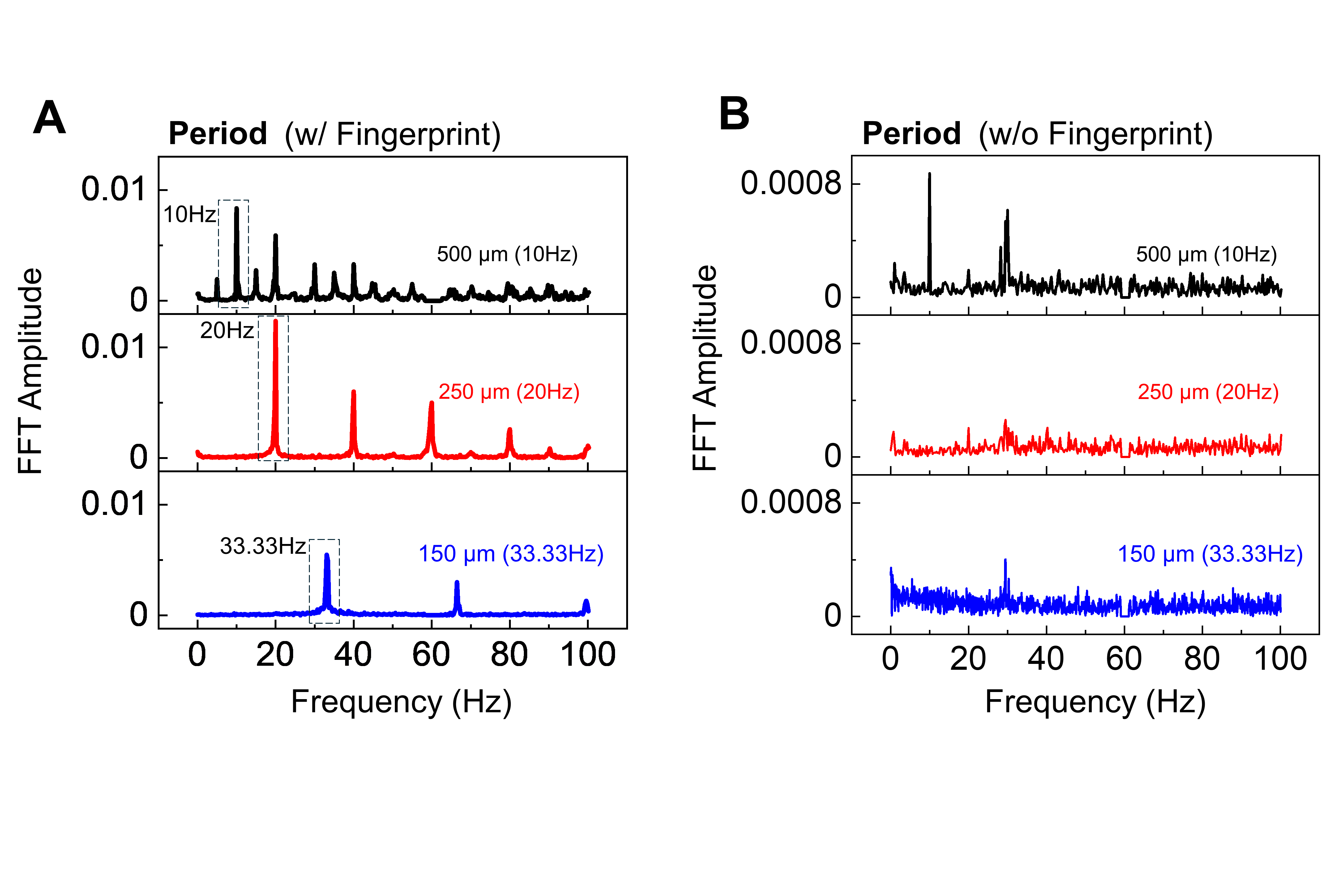


**Figure S15.** Frequency-domain analysis of Meissner sensor responses depending on fingerprint structure and grating period. (A and B) Fast Fourier transform (FFT) spectra for gratings with varying periods (500, 250, and 150 μm) under a constant height of 300 μm. The case with fingerprint structure exhibits pronounced peaks at the imposed frequencies (A), while the sensor without fingerprints shows diminished amplitudes and fails to identify the applied frequencies as the period decreases (B).


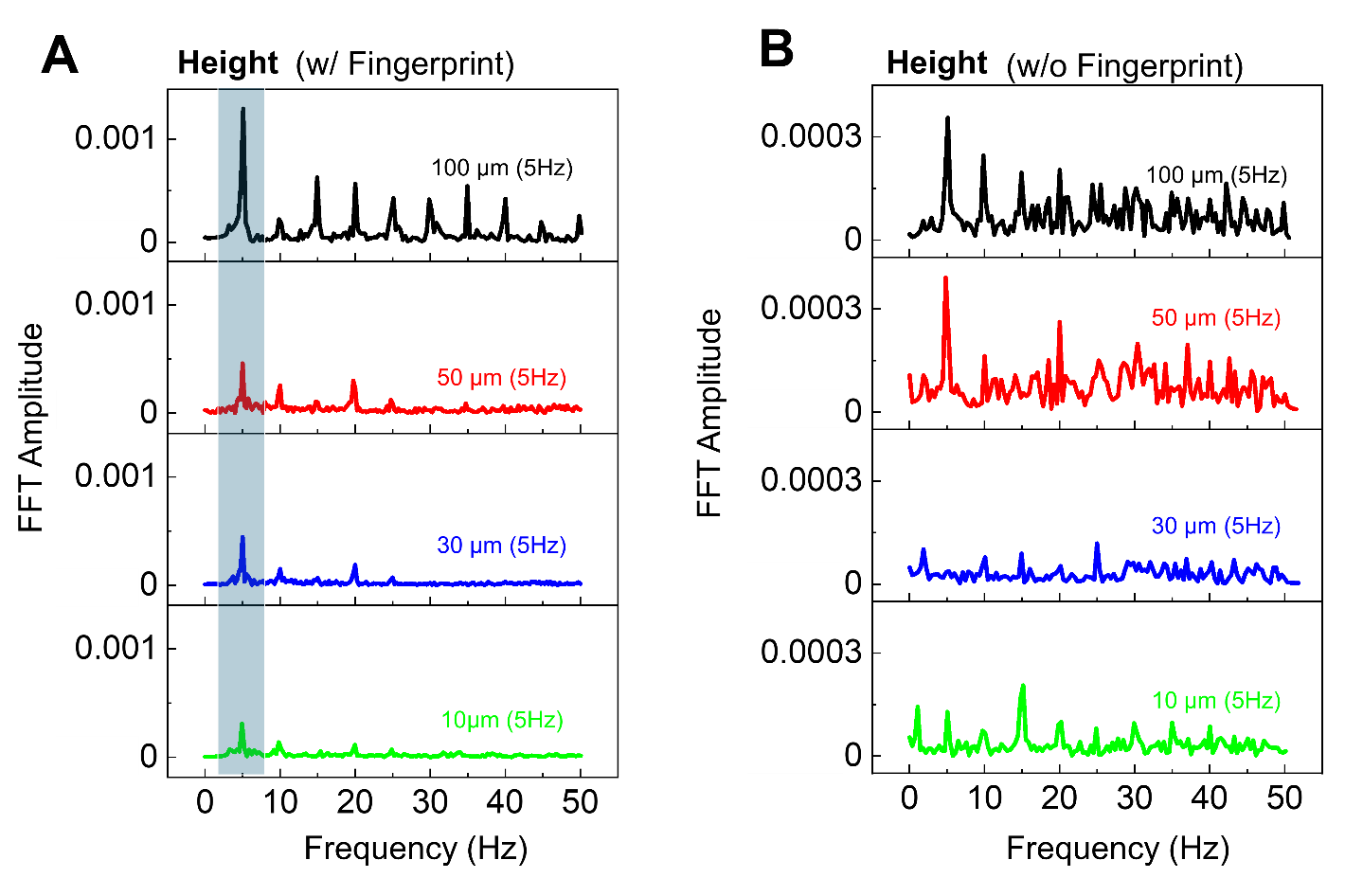


**Figure S16.** Frequency-domain analysis of Meissner sensor responses depending on fingerprint structure and grating heights. (A and B) FFT spectra for gratings with different heights (10, 30, 50, and 100 μm) at a fixed period of 1 mm. In the presence of fingerprint structures, distinct frequency peaks are maintained even at small grating heights (A), whereas the fingerprint-free sensor shows weak and unstable responses (B).


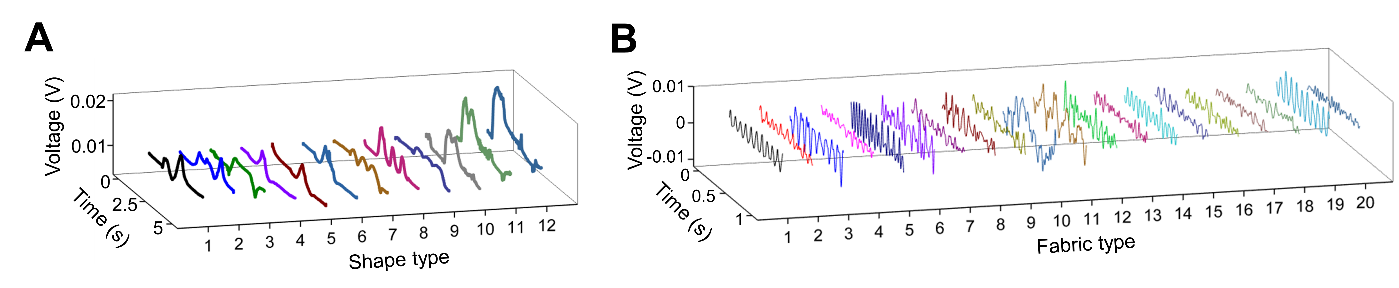


**Figure S17.** Raw time-domain signals for shape and fabric recognition experiments. (A) Representative raw time-domain voltage signals obtained from the Merkel sensor during sliding over twelve different geometric shapes. The Merkel sensor generated distinct waveform profiles depending on the shape, reflecting differences in macroscopic geometry and curvature. (B) Representative raw time-domain voltage signals obtained from the Meissner sensor during sliding over twenty different fabrics. The Meissner sensor produced vibration-rich signals corresponding to the microscopic texture and periodic surface features of each fabric. These raw signals were converted into STFT spectrograms and used for CNN-based classification and t-SNE-based feature visualization in Figure 5.


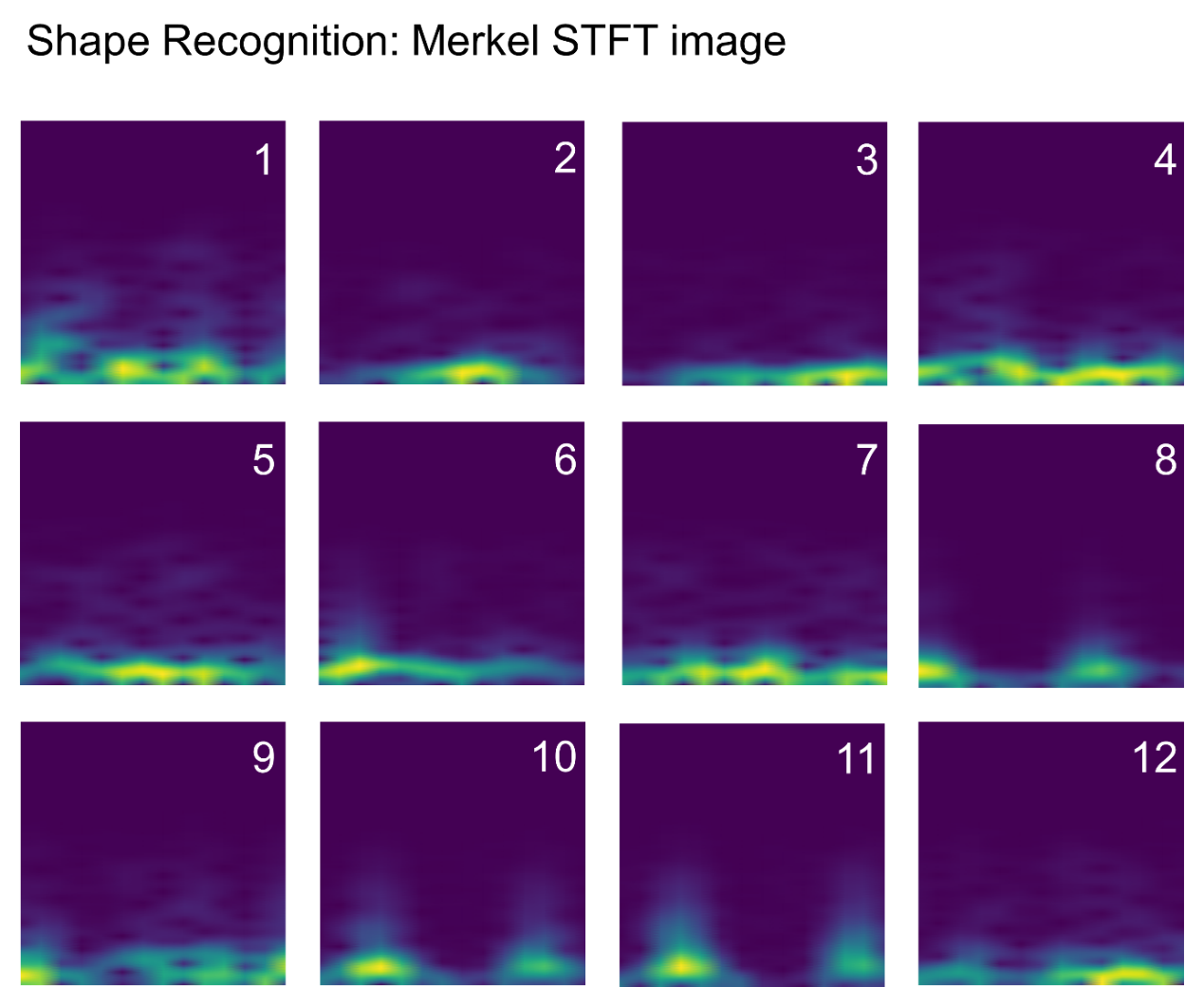


**Figure S18.** STFT spectrogram datasets used for CNN classification and t-SNE analysis.

STFT spectrograms of Merkel sensor signals for shape recognition.


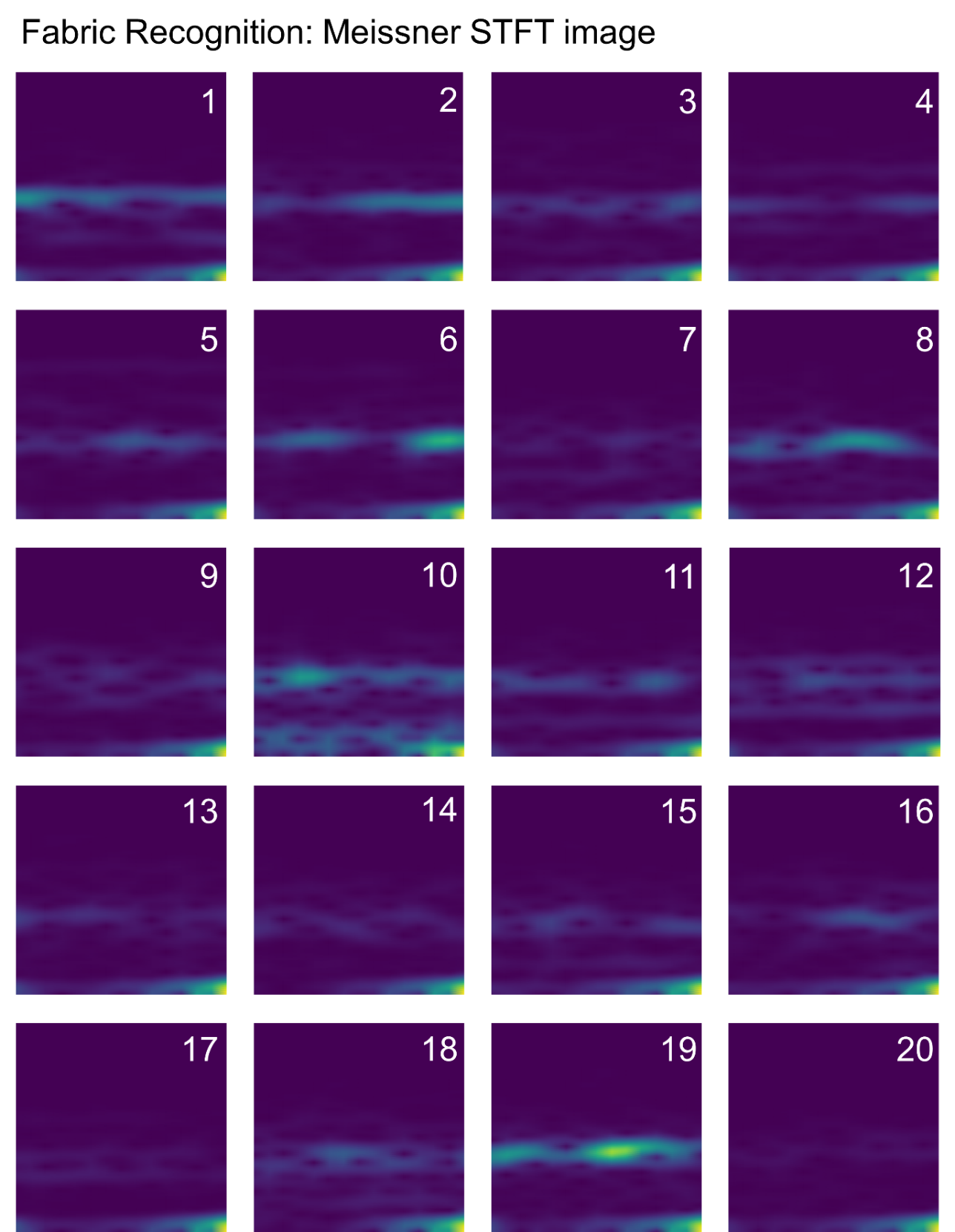


**Figure S19.** STFT spectrogram datasets used for CNN classification and t-SNE analysis. STFT spectrograms of Meissner sensor signals for fabric recognition.


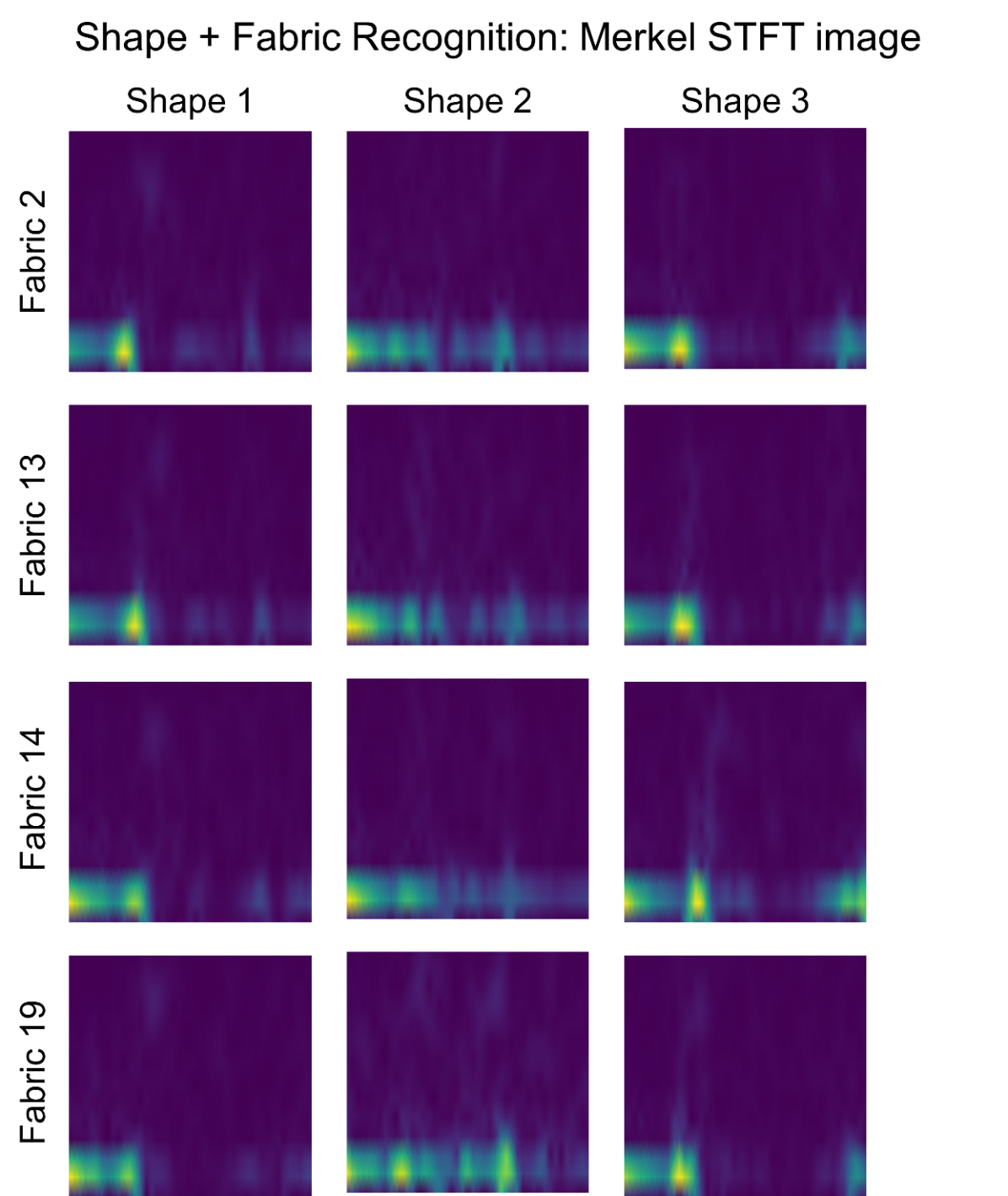


**Figure S20.** STFT spectrogram datasets used for CNN classification and t-SNE analysis. STFT spectrograms of Merkel sensor signals for combined shape–fabric recognition.


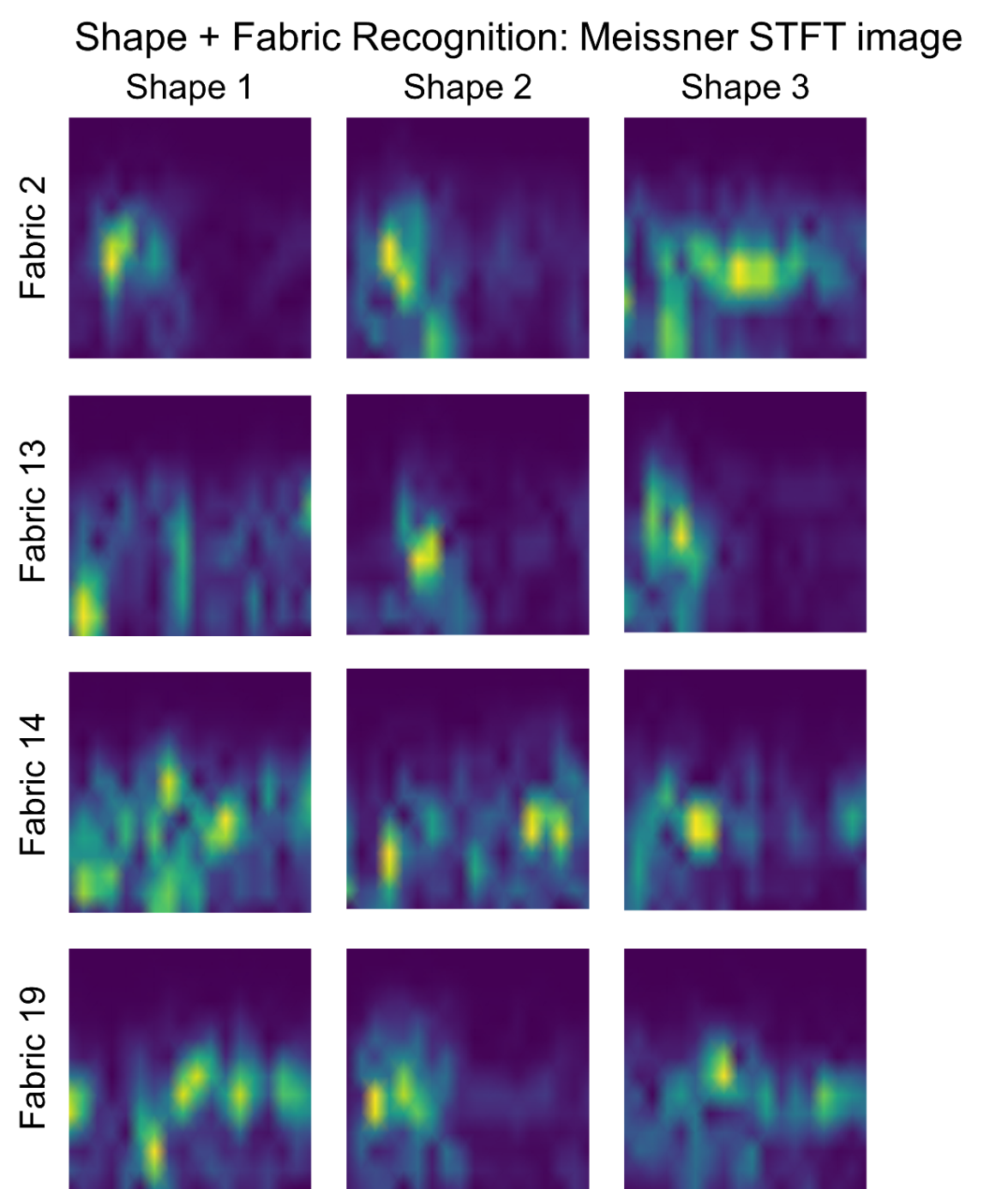


**Figure S21.** STFT spectrogram datasets used for CNN classification and t-SNE analysis. STFT spectrograms of Meissner sensor signals for combined shape–fabric recognition.


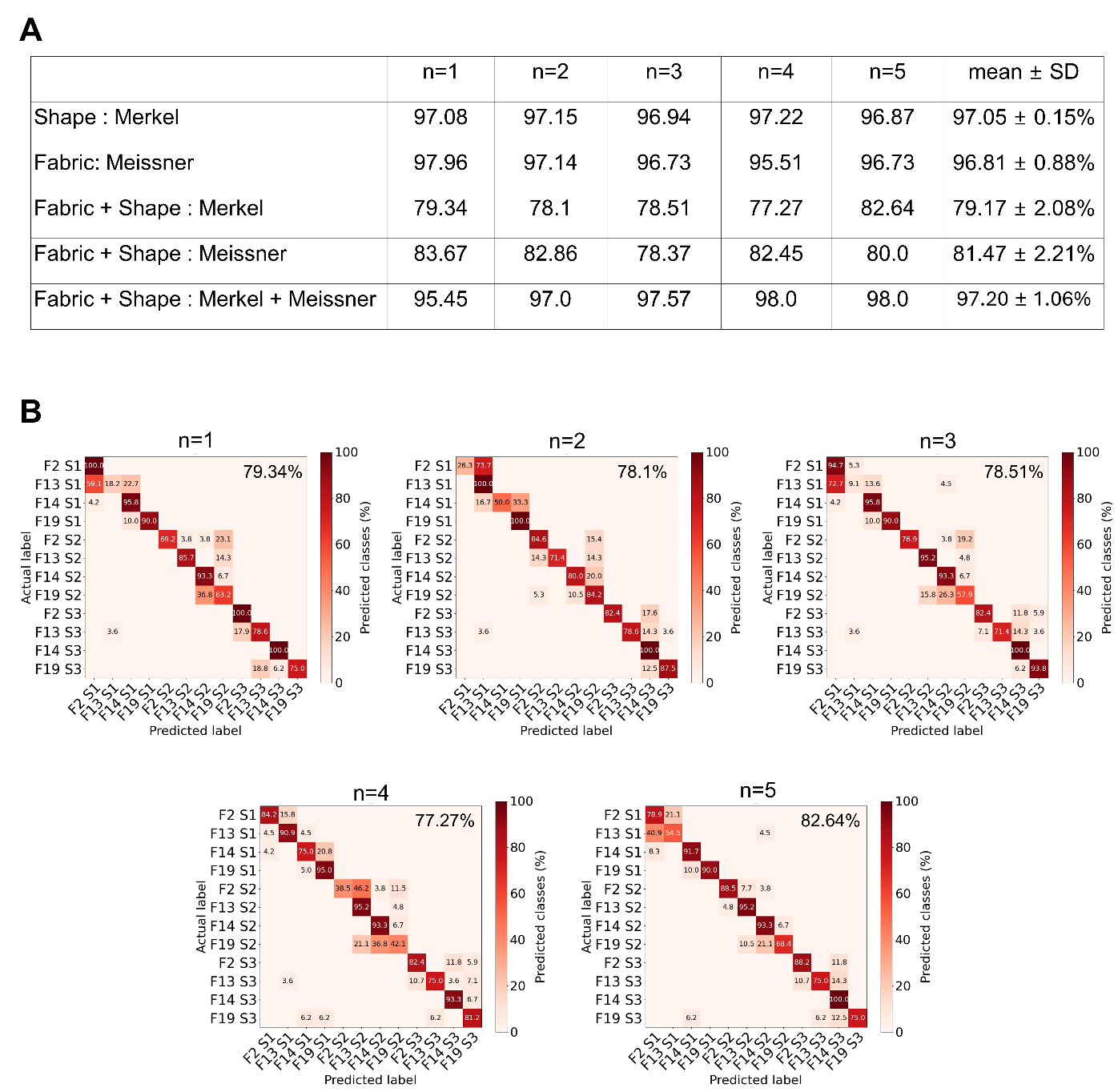


**Figure S22.** Inter-device reproducibility of CNN-based tactile classification using five independently fabricated sensors. (A) Classification accuracies obtained from five independently fabricated sensors for shape recognition using the Merkel sensor, fabric recognition using the Meissner sensor, fabric–shape recognition using the Merkel sensor, fabric–shape recognition using the Meissner sensor, and fabric–shape recognition using combined Merkel and Meissner sensor data. The results are summarized as mean ± SD. (B) Confusion matrices for fabric–shape recognition using only the Merkel sensor from five independently fabricated sensors. Across all devices, the Merkel sensor consistently classified shape-dependent features while showing limited discrimination among different fabric classes within the same shape category, indicating its modality-specific contribution to shape recognition.


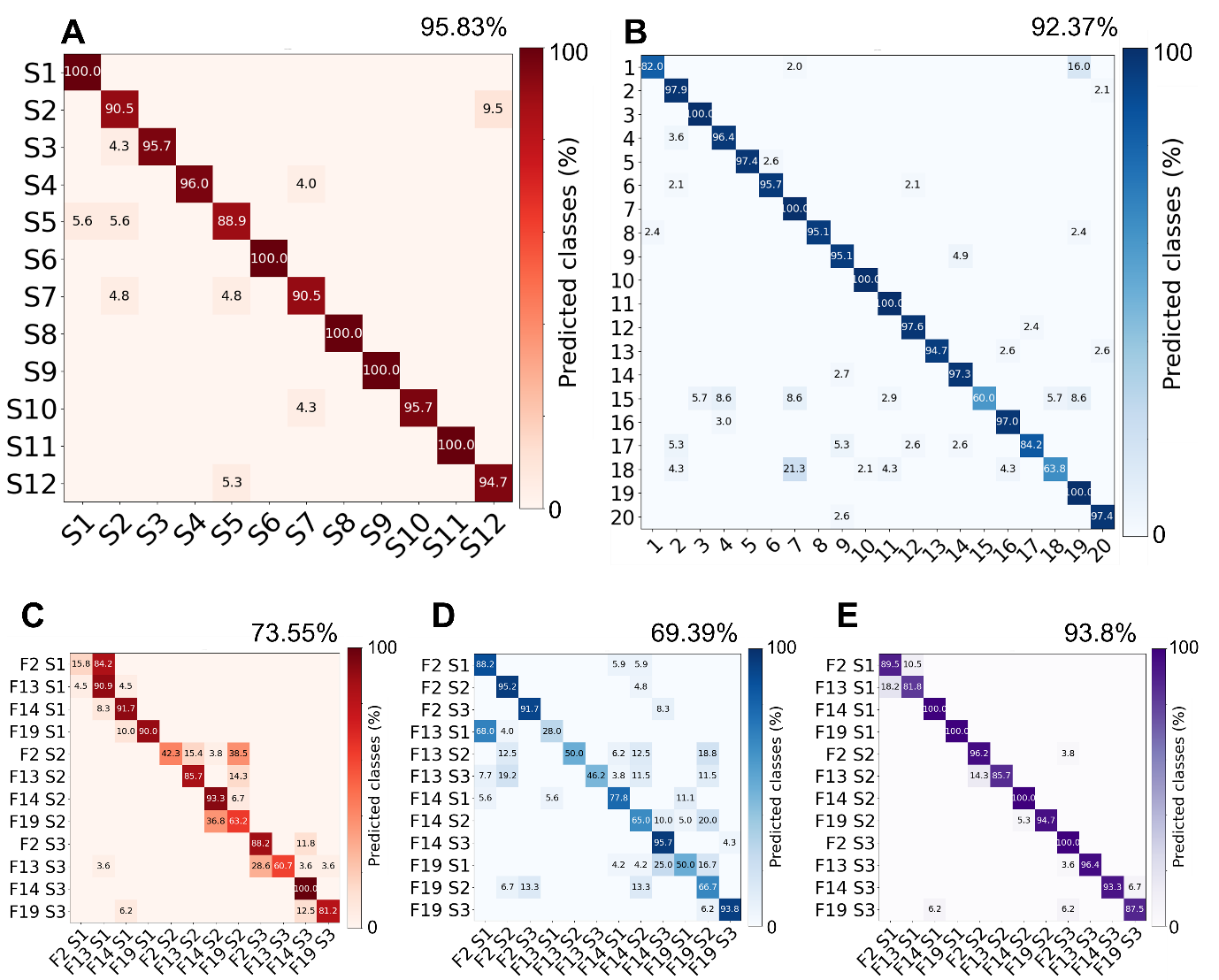


**Figure S23.** Classification performance of the sensor without papillary structure under the same CNN architecture. CNN classification results obtained from the sensor without the papillary structure while keeping the data-processing pipeline, STFT conversion, and CNN architecture identical to those used for the papillary-structured sensor. (A) Shape recognition using Merkel sensor data only, showing an accuracy of 95.83%. (B) Fabric recognition using Meissner sensor data only, showing an accuracy of 92.37%. (C) Fabric–shape recognition using Merkel sensor data only, showing an accuracy of 73.55%. (D) Fabric–shape recognition using Meissner sensor data only, showing an accuracy of 69.39%. (E) Fabric–shape recognition using combined Merkel and Meissner sensor data, showing an accuracy of 93.8%. Compared with the papillary-structured sensor, the non-papillary sensor exhibited lower accuracy in all tasks, with a more pronounced decrease in Meissner-related fabric and fabric–shape recognition. This result indicates that the papillary structure improves the quality of slip-induced dynamic features delivered to the Meissner sensor, consistent with the clearer periodic slip responses observed in Figure 3E. The dual-channel configuration still outperformed each single-channel configuration, confirming the complementary contribution of Merkel and Meissner signals to multimodal recognition.


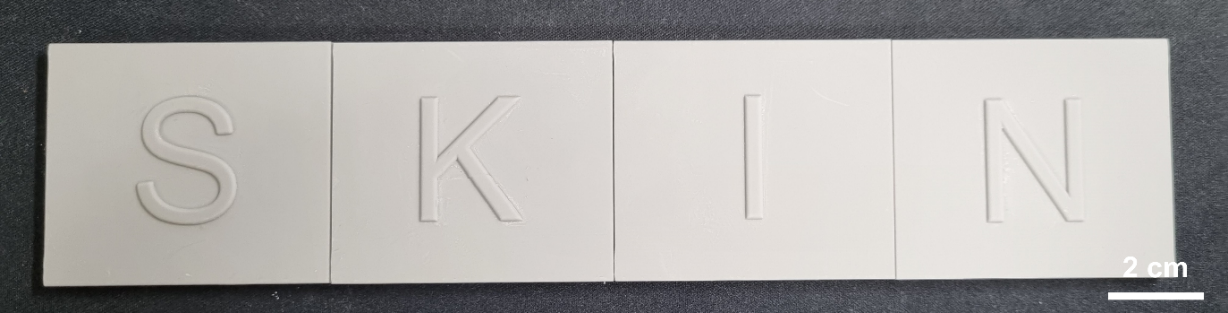


**Figure S24.** Photograph of the embossed “SKIN” molds used in the tactile regeneration experiments shown in Figure 6. Mold features raised letters (embossed height ≈ 1 mm) fabricated by 3D printing.
